# The associations of longitudinal changes in consumption of total and types of dairy products and markers of metabolic risk and adiposity: findings from the European Investigation into Cancer and Nutrition (EPIC)–Norfolk study, United Kingdom

**DOI:** 10.1093/ajcn/nqz335

**Published:** 2020-01-08

**Authors:** Eirini Trichia, Robert Luben, Kay-Tee Khaw, Nicholas J Wareham, Fumiaki Imamura, Nita G Forouhi

**Affiliations:** 1 Medical Research Council Epidemiology Unit, Institute of Metabolic Science, University of Cambridge School of Clinical Medicine, Cambridge, United Kingdom; 2 Department of Public Health and Primary Care, Institute of Public Health, University of Cambridge School of Clinical Medicine, Cambridge, United Kingdom

**Keywords:** dairy products, milk, yogurt, cheese, butter, cardiometabolic disease, adiposity, analysis of change, adults

## Abstract

**Background:**

The consumption of some types of dairy products has been associated with lower cardiometabolic disease incidence. Knowledge remains limited about habitual dairy consumption and the pathways to cardiometabolic risk.

**Objective:**

We aimed to investigate associations of habitual consumption of total and types of dairy products with markers of metabolic risk and adiposity among adults in the United Kingdom.

**Methods:**

We examined associations of changes in dairy consumption (assessed with a food-frequency questionnaire) with parallel changes in cardiometabolic markers using multiple linear regression among 15,612 adults aged 40–78 y at baseline (1993–1997) and followed up over 1998–2000 (mean ± SD: 3.7±0.7 y) in the European Prospective Investigation into Cancer and Nutrition (EPIC)–Norfolk study.

**Results:**

For adiposity, an increase in fermented dairy products [yogurt (total or low-fat) or low-fat cheese] consumption was associated with a lower increase in body weight and body mass index (BMI). For example, over 3.7 y, increasing yogurt consumption by 1 serving/d was associated with a smaller increase in body weight by 0.23 kg (95% CI: −0.46, −0.01 kg). An increase in full-fat milk, high-fat cheese, and total high-fat dairy was associated with greater increases in body weight and BMI [e.g., for high-fat dairy: β = 0.13 (0.05, 0.21) kg and 0.04 (0.01, 0.07) kg/m^2^, respectively]. For lipids, an increase in milk (total and low-fat) or yogurt consumption was positively associated with HDL cholesterol. An increase in total low-fat dairy was negatively associated with LDL cholesterol (−0.03 mmol/L; −0.05, −0.01 mmol/L), whereas high-fat dairy (total, butter, and high-fat cheese) consumption was positively associated [e.g., 0.04 (0.02, 0.06) mmol/L for total high-fat dairy]. For glycemia, increasing full-fat milk consumption was associated with a higher increase in glycated hemoglobin (*P* = 0.027).

**Conclusions:**

The habitual consumption of different dairy subtypes may differently influence cardiometabolic risk through adiposity and lipid pathways.

## Introduction

It is of public health interest for formulating dietary guidelines to understand the link between consumption of different types of dairy products and health, but current scientific evidence remains controversial, with heterogeneous prospective associations with cardiometabolic diseases (1–3). The mechanisms for positive or inverse associations by dairy type remain largely unknown, although several relevant intermediate metabolic endpoints have been examined. The most studied intermediate endpoints are markers of adiposity including body weight, body fat, and lean mass. Specifically, dairy consumption decreased body weight and body fat mass in randomized controlled trials that applied energy restriction in both dairy and nondairy arms (4–7). Conversely, trials of dairy products with or without energy restriction reported increased body lean mass (5–7). Most of these intervention trials were of short duration (on average, 7.7 mo) and used mixed dairy products, precluding distinction between effects of specific dairy types (6). Evidence on associations of different types of dairy products with other intermediate markers is sparse for glycemia, inflammation, blood pressure, and lipids, although there is evidence that butter consumption elevates total cholesterol, LDL cholesterol, and HDL cholesterol (8, 9).

In general populations, evidence for long-term habitual dairy consumption is heterogeneous and inconclusive (10). In observational prospective studies, associations of changes in dietary factors with changes in metabolic markers during the same period (parallel change) may show less-biased associations, closer to associations observed in randomized controlled trials (11). However, only a few studies adopted such analyses of dairy consumption (10), especially for dairy types (12–14). Therefore, we aimed to investigate associations of changes in total and types of dairy product consumption with parallel changes in markers of metabolic risk and adiposity, as potential pathways for the association of dairy products with cardiometabolic disease.

## Methods

### Study design and population

We evaluated data from the European Prospective Investigation into Cancer and Nutrition–Norfolk (EPIC-Norfolk) study in the United Kingdom (15). At baseline (1993–1997; first health check), 25,639 participants without a terminal or malignant disease or inability to attend a local clinic, alcoholism, psychiatric disorder, inadequate command of English, or blindness were recruited through general practices. They attended a first follow-up (1998–2000; second health check) and a second follow-up (2004–2011; third health check). We included 15,612 adults after the following exclusions: 8507 adults who did not undergo follow-up assessments, 673 adults without dietary data, and 847 adults with extreme values of dietary intakes based on total energy intake [<800 and >4000 kcal/d for men and <500 and >3500 kcal/d for women (16)] or extreme changes in dairy consumption or cardiometabolic marker for each association examined (outside the range of 3 SDs from the mean). **Supplemental Figure 1** shows the flowchart of participant selection. Informed consent was obtained from each participant, and the study was approved by the Norwich District Ethics Committee.

### Dietary assessment

Habitual diet was assessed at baseline and follow-up with a 130-item semiquantitative food-frequency questionnaire (FFQ). Internal validity of the questionnaire was assessed against 7-d food diaries (17). The questionnaire ascertained habitual consumption of dairy products over the past year with 9 frequencies ranging from “never or less than once/month” to “six times per day” and included additional questions on the type and amount of milk consumed. The correlation coefficients for dairy products between the questionnaire and the 7-d diary at baseline were 0.56 for milk, 0.57 for yogurt, 0.33 for cheese, and 0.54 for butter. We processed dietary data with the FETA software (18), and expressed them in servings/day. We considered total and types of dairy products as well as their low- and high-fat subtypes and grouped them as shown in **Table 1** (19).

**TABLE 1:** Classification of dairy products assessed with food-frequency questionnaires: the EPIC-Norfolk study^1^

Dairy group	Food items queried in the food-frequency questionnaire
Full-fat milk	Goat's milk; Channel Islands milk; silver top full-cream milk; evaporated milk, whole diluted; sheep's milk
Low-fat milk	Semi-skimmed milk, skimmed milk, skimmed milk as reconstituted dried milk
Milk	Full-fat milk, low-fat milk
Yogurt	Full-fat yogurt,^2^ low-fat yogurt^2^
Cheese	High-fat cheese,^3^ low-fat cheese^4^
Cream^5^	Single cream, double cream
Low-fat fermented dairy products	Yogurt, low-fat cheese
Fermented dairy products	Yogurt, cheese
High-fat dairy products (≥3.9% fat)	Full-fat milk, high-fat cheese, cream, butter, ice cream
Low-fat dairy products (<3.9% fat)	Low-fat milk, yogurt, low-fat cheese
Total dairy products	Milk, yogurt, cheese, cream, butter, ice cream

1 EPIC, European Prospective Investigation into Cancer and Nutrition.

2 The variables derived directly from the questions in the food-frequency questionnaire were used.

3 The variable derived directly from the questions in the food-frequency questionnaire on hard cheese intake was used. The assumption made here is that high-fat cheese is equivalent to hard cheese.

4 The variable derived directly from the questions in the food-frequency questionnaire on cottage and low-fat soft cheese intake was used. This was under the assumption that low-fat cheese would be equivalent to cottage and low-fat, soft cheese.

5 Cream was used as a contributor to high-fat and total dairy products, but results separately for it and its types are not presented, as the very low intakes result in very unstable and imprecise estimates.

### Assessment of markers of metabolic risk and adiposity

We examined changes in BMI, waist circumference, the ratio of total to HDL cholesterol, glycated hemoglobin (HbA1c), and blood pressure (systolic and diastolic) as primary outcomes. Changes in weight, waist-to-hip ratio, total cholesterol, HDL cholesterol, LDL cholesterol, triglycerides, and a metabolic-risk *z* score were examined as secondary outcomes. Weight, height, waist circumference, and hip circumference were measured with a standardized protocol. BMI was defined as weight divided by height squared. Blood pressure was measured twice (using the average) with an Accutorr sphygmomanometer (Datascope). Total cholesterol, HDL cholesterol, triglycerides (RA 1000; Bayer Diagnostics) and HbA1c (Diamat ion exchange HPLC; Bio-Rad Laboratories) were measured in nonfasting blood. LDL cholesterol was calculated using the Friedewald formula (20). The metabolic risk *z* score was calculated as previously described (21) by averaging *z* scores of blood pressure (systolic and diastolic), HbA1c, waist circumference, HDL cholesterol multiplied by −1, and log-transformed triglycerides.

### Assessment of sociodemographic and lifestyle factors

Sociodemographic factors, medical history, and smoking status were assessed with the Health and Lifestyle Questionnaire at baseline and follow-up (22). Physical activity at baseline was assessed with the same questionnaire, but at follow-up it was assessed with the EPIC physical activity questionnaire (EPAQ2) designed based on validation studies using heart rate monitoring after calibration with estimates derived from doubly-labeled water (23).

### Statistical analyses

Triglycerides and the ratio of total to HDL cholesterol were not normally distributed and were log-transformed. Missing values of the dairy exposures and the covariates were imputed using multiple imputation by chained equations whereby we generated and analyzed 5 imputed datasets (24).

We investigated the association of the change in dairy consumption between baseline and the first follow-up with the parallel change of the markers using multiple linear regression models. This approach was previously indicated to give results comparable to those from randomized controlled trials (11). As a positive control analysis, we first evaluated the prospective association of changes in butter consumption with changes in LDL cholesterol, which were expected to be positive from evidence from randomized controlled trials (8).

We controlled for potential confounders in 3 regression models. The first model included age, sex, educational level, age at completion of full-time education, type of occupation, marital status, physical activity level, smoking status, total energy intake, medications [lipid-lowering, antihypertensive, and hormone replacement therapy (in women only)], and follow-up duration. The second model additionally included consumption of fruit, vegetables, potatoes, legumes, nuts, processed cereals, whole-grain cereals, poultry and eggs, red meat, processed meat, fish, sauces, margarine, sweet snacks, sugar-sweetened beverages, artificially sweetened beverages, fruit juice, coffee, tea, and alcoholic beverages and dietary supplement use. The third model additionally included BMI unless the outcome was BMI or the metabolic-risk *z* score. Models included baseline values of covariates and their changes if applicable. Dairy types were mutually adjusted for in each model. Baseline values of each outcome were not adjusted for to avoid collider bias (25–27).

Interactions with age, sex, and BMI were examined. We also conducted analyses to assess potential bias due to healthy survivor effect and censoring over the follow-up, applying inverse probability weighting after deriving weights of censoring with logistic regression (28). To assess stability of results, we repeated analyses with 10 imputed datasets and a complete-case dataset; analyses excluding participants with prevalent type 2 diabetes and additionally with hypertension, hyperlipidemia, or cardiovascular disease; and analyses adjusting for the baseline value of the outcome under study. In secondary analyses, we tested the same hypotheses with a linear mixed model predicting repeated measures of outcomes (first and second follow-up) by repeated measures of dairy consumption (baseline and first follow-up) (hereafter, referred to as the “secondary longitudinal analysis”). Associations were considered statistically significant at the nominal level (α = 0.05). For all analyses we used Stata 14.2 (StataCorp LP, 2015).

## Results

### Descriptive characteristics

Participants were followed for a mean ± SD of 3.7 ± 0.7 y. The mean ± SD change in the estimated consumption of dairy products was −0.06 ± 0.71 servings/d for milk, 0.02 ± 0.41 for yogurt, and −0.04 ± 0.38 for cheese (**Table 2**). Baseline values and changes in physiological markers and lifestyle characteristics by dairy products are presented in **Table 3** and **Supplemental Tables 1** and **2**.

**TABLE 2:** Descriptive characteristics of total and types of dairy products at baseline, first follow-up, and the change between baseline and first follow-up in the EPIC-Norfolk study^1^

	Dairy products, servings/d		
	Baseline	First follow-up	Change
Milk			
Total	1.73 ± 0.82	1.67 ± 0.82	−0.06 ± 0.71
Full-fat	0.33 ± 0.78	0.23 ± 0.66	−0.11 ± 0.62
Low-fat	1.39 ± 1.00	1.43 ± 0.95	0.04 ± 0.86
Yogurt			
Total	0.30 ± 0.41	0.33 ± 0.42	0.02 ± 0.41
Full-fat	0.04 ± 0.12	0.04 ± 0.14	0.00 ± 0.16
Low-fat	0.27 ± 0.39	0.29 ± 0.41	0.02 ± 0.40
Cheese			
Total	0.47 ± 0.40	0.43 ± 0.37	−0.04 ± 0.38
High-fat	0.34 ± 0.29	0.30 ± 0.27	−0.03 ± 0.28
Low-fat	0.13 ± 0.27	0.12 ± 0.26	0.00 ± 0.28
Fermented dairy products	0.77 ± 0.61	0.76 ± 0.61	−0.01 ± 0.57
Cream	0.07 ± 0.17	0.07 ± 0.18	0.00 ± 0.18
Butter	0.43 ± 0.93	0.44 ± 0.89	0.00 ± 0.88
Ice cream	0.21 ± 0.28	0.20 ± 0.30	−0.01 ± 0.30
Total dairy products			
Total	3.22 ± 1.41	3.15 ± 1.39	−0.07 ± 1.32
High-fat	1.18 ± 1.41	1.05 ± 1.29	−0.14 ± 1.18
Low-fat	1.82 ± 1.18	1.89 ± 1.14	0.06 ± 1.02

1 Values are means ± SDs, estimated from 15,612 adults. The duration between the baseline and the first follow-up was 3.7 y on average. Dairy consumption was assessed with a food-frequency questionnaire.

EPIC, European Prospective Investigation into Cancer and Nutrition.

**TABLE 3:** Baseline descriptive statistics of sociodemographic, behavioral, clinical, and dietary factors by the change in milk, yogurt, and cheese consumption: the EPIC-Norfolk study^1^

	Total baseline	Changes in milk		Changes in yogurt		Changes in cheese	
Servings/d, mean ± SD change	—	−0.06 ± 0.71		0.02 ± 0.41		−0.04 ± 0.38	
Change range^2^	—	−3.7, −0.7	0.7, 3.7	−5.1, −0.1	0.2, 5.0	−4.3, −0.2	0.2, 4.4
Participants,^3^*n*	15,612	2050	3181	2540	3030	2818	2942
Sociodemographic factors							
Age, y	58.6 ± 8.9	58.3 ± 8.9	59.1 ± 8.9	58.3 ± 8.5	58.4 ± 8.8	58.6 ± 9.0	58.9 ± 8.9
Female sex,^4^ %	56.2	53.8	56.6	64.7	63.3	61.0	58.7
Educational level,^4^ %							
Medium	41.9	42.0	40.2	41.7	41.1	42.4	40.9
High	14.7	14.0	12.7	15.8	15.7	16.3	14.9
Socioeconomic status,^4^ %							
Medium	16.7	16.7	16.3	18.3	16.1	17.0	16.3
High	46.7	45.7	44.5	48.7	49.5	48.2	46.4
Marital status,^5^ %							
Married	82.6	82.5	83.6	80.9	82.1	80.0	83.7
Widowed/separated	13.4	13.8	12.2	15.3	14.0	15.6	12.6
Lifestyle factors							
Smoking status, %							
Former	41.4	43.5	42.2	39.2	40.5	43.0	41.3
Current	9.4	10.3	10.0	6.6	7.3	8.3	7.7
Physical activity,^4^, ^6^ %							
Moderately inactive	29.6	28.2	29.7	31.7	30.2	29.7	30.2
Moderately active	24.3	24.4	24.1	25.6	26.0	24.0	24.6
Active	19.3	20.9	19.5	19.5	18.9	19.9	19.2
Energy intake,^6^ kJ/d	8443 ± 2310	8535 ± 2259	8209 ± 2293	8548 ± 2209	8389 ± 2318	8774 ± 2326	8293 ± 2301
Medications/supplements, %							
Lipid-lowering medication^4^	1.5	1.7	1.4	2.0	1.6	1.1	2.0
Antihypertensive medication^4^	16.4	17.2	17.1	16.1	16.7	16.5	17.7
Hormonal therapy^4^	12.3	12.2	11.9	16.1	14.2	13.6	12.5
Dietary supplements^4^, ^6^	49.6	51.2	50.5	44.2	44.9	50.6	48.2
Nondairy food dietary factors,^6^ g/d							
Fruits	250.2 ± 180.4	240.1 ± 171.1	256.5 ± 194.6	286.1 ± 186.6	272.2 ± 191.4	275.4 ± 197.5	256.2 ± 177.4
Vegetables	241.8 ± 124.0	234.4 ± 122.3	245.8 ± 124.3	264.6 ± 126.3	248.0 ± 131.4	260.5 ± 138.8	245.6 ± 128.2
Potatoes	115.2 ± 60.5	115.7 ± 67.1	114.9 ± 57.7	111.3 ± 57.5	112.6 ± 61.3	115.4 ± 64.0	114.2 ± 56.4
Legumes	60.0 ± 37.6	59.9 ± 38.1	60.7 ± 36.1	62.1 ± 39.9	59.6 ± 37.2	62.1 ± 39.8	60.8 ± 37.4
Processed cereals	82.1 ± 54.1	83.6 ± 55.4	79.2 ± 55.6	81.7 ± 56.0	80.4 ± 50.9	82.7 ± 52.4	81.2 ± 53.1
Whole-grain cereals	78.9 ± 78.0	77.6 ± 76.9	77.6 ± 79.6	91.8 ± 81.1	84.3 ± 80.5	86.7 ± 79.6	76.5 ± 75.0
Poultry and eggs	37.9 ± 23.9	36.6 ± 23.1	38.3 ± 24.4	39.8 ± 25.4	37.9 ± 22.1	38.1 ± 26.7	37.1 ± 23.2
Red meat	62.0 ± 40.5	61.4 ± 44.5	62.0 ± 40.7	59.5 ± 37.9	61.1 ± 39.2	61.5 ± 40.6	61.4 ± 41.2
Processed meat	27.9 ± 22.9	27.7 ± 22.6	28.5 ± 23.2	25.1 ± 20.5	27.3 ± 21.9	26.8 ± 23.8	27.5 ± 22.6
Fish	37.7 ± 25.8	36.4 ± 26.3	37.7 ± 25.5	40.3 ± 27.6	38.6 ± 26.0	38.9 ± 27.2	37.0 ± 26.2
Sauces	19.5 ± 17.9	19.2 ± 17.4	19.1 ± 17.3	20.3 ± 17.0	19.2 ± 16.7	21.0 ± 18.5	19.4 ± 18.4
Margarine	16.6 ± 16.3	16.6 ± 17.0	16.0 ± 16.1	16.2 ± 15.1	16.3 ± 15.9	17.2 ± 16.4	16.6 ± 16.2
Nuts	2.5 ± 7.4	2.6 ± 7.9	2.5 ± 7.3	2.7 ± 7.5	2.1 ± 5.2	3.0 ± 8.5	2.4 ± 6.8
Sweet snacks	116.6 ± 84.6	113.7 ± 81.8	116.0 ± 86.2	106.8 ± 76.5	115.7 ± 83.8	114.9 ± 83.3	115.9 ± 86.5
Sugar-sweetened beverages	33.1 ± 72.1	33.6 ± 75.3	33.6 ± 75.9	29.0 ± 59.2	33.8 ± 77.6	34.8 ± 74.1	30.4 ± 63.9
Artificially sweetened beverages	36.9 ± 104.9	34.4 ± 99.1	38.5 ± 110.1	43.0 ± 110.7	41.5 ± 110.7	44.9 ± 120.6	34.6 ± 100.2
Fruit juice	50.9 ± 69.1	49.6 ± 72.9	50.9 ± 69.1	57.1 ± 66.5	55.6 ± 73.7	53.2 ± 67.6	52.4 ± 71.6
Regular coffee	329.5 ± 320.6	319.7 ± 320.8	325.7 ± 320.1	321.1 ± 309.4	329.7 ± 317.9	343.4 ± 328.2	325.3 ± 318.3
Decaffeinated coffee	87.6 ± 207.1	86.0 ± 208.6	86.6 ± 205.0	101.0 ± 216.2	103.3 ± 221.1	94.4 ± 214.9	93.7 ± 215.9
Tea	632.0 ± 365.2	659.8 ± 365.9	630.7 ± 362.1	622.8 ± 362.1	626.6 ± 354.2	613.8 ± 371.6	635.5 ± 365.2
Alcoholic beverages	128.8 ± 232.2	131.6 ± 251.1	122.1 ± 227.5	109.0 ± 180.3	115.1 ± 210.4	125.7 ± 222.2	119.1 ± 223.6

1 Continuous variables are presented as means ± SDs and categorical variables are presented as column percentages. EPIC, European Prospective Investigation into Cancer and Nutrition.

2 The 2 extreme categories are the first and fifth quintile of the change in consumption of milk, yogurt, and cheese from baseline to the first follow-up

3 Total percentage of missing values: 13% at baseline, 59% (49% due to missing values of the physical activity variable) at follow-up, and 60% at both when accounting for nonoverlapping missing values for all of the variables.

4 One category is not shown for succinctness: sex, men; educational level, low; socioeconomic status, low; physical activity, inactive; lipid-lowering medication, no; antihypertensive medication, no; hormonal therapy, no; dietary supplements, no.

5 Missing values <5% at baseline and follow-up.

6 Missing values <5% at baseline, but 20–50% at follow-up.

### Dairy products and anthropometric markers

**FIGURE 1:**
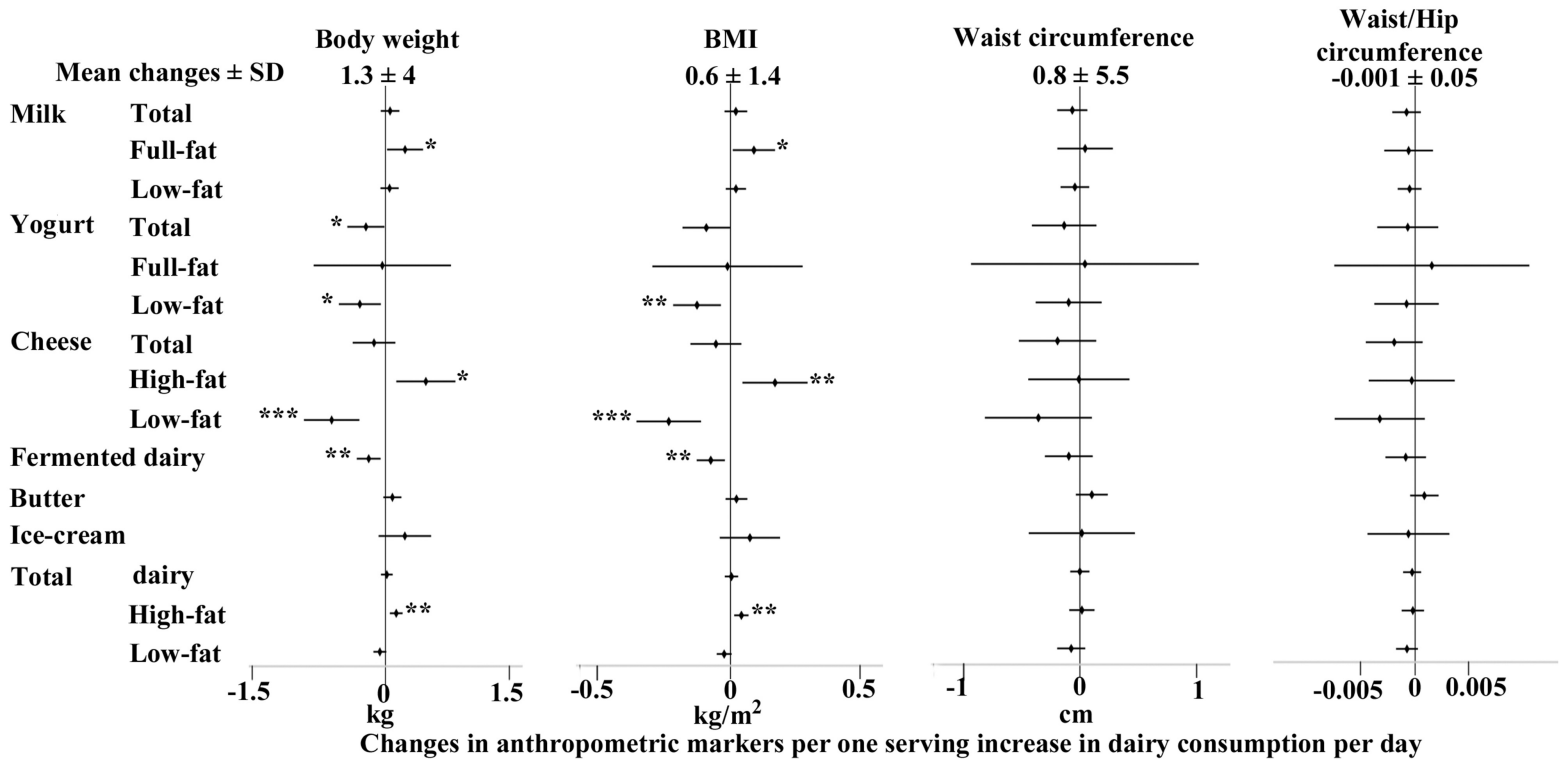
Associations of increases in dairy consumption (by 1 serving/d) with changes in anthropometric markers (body weight: *n* = 14,044; BMI: *n* = 14,134; waist circumference: *n* = 14,227; waist-to-hip ratio: *n* = 14,213) over an average of 3.7 y of follow-up. Mean ± SD changes presented in the top heading represent the average change in the markers over the follow-up. Forest plots represent linear regression coefficients with their 95% CIs adjusted for sociodemographic (age, sex, education, socioeconomic status), lifestyle (physical activity, smoking status), clinical (medication use, BMI), and dietary factors (total energy intake, food groups). *P < 0.05, **P < 0.01, ***P < 0.001.

Body weight and BMIincreased by 1.3 ± 4.0 kg and 0.6 ± 1.4 kg/m^2^, respectively, over the follow-up. An increase in each of fermented dairy products, yogurt (total and low-fat), or low-fat cheese was associated with a smaller increase in body weight and BMI. For example, those who increased their yogurt consumption by 1 serving/d had a lower increase in body weight by 0.23 kg (95% CI: −0.46, −0.01 kg) in comparison to those who did not change yogurt consumption (**Figure 1**, **Supplemental Table 3**). An increase in full-fat milk, high-fat cheese, and total high-fat dairy consumption was associated with a higher increase in body weight by, for example, 0.13 kg per 1 serving of total high-fat dairy/d (95% CI: 0.05, 0.21 kg; Figure 1).We observed similar associations for full-fat milk and high-fat cheese. Changes in total dairy products or other dairy types were not significantly associated with changes in body weight or BMI. Changes in any dairy types were not significantly associated with changes in waist circumference or waist-to-hip ratio (Figure 1).

### Dairy products and metabolic markers

**FIGURE 2:**
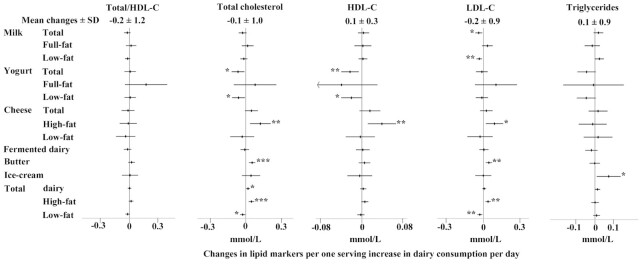
Associations of increases in dairy consumption (by 1 serving/d) with changes in lipid markers (total-to-HDL cholesterol: *n* = 12,959; total cholesterol: *n* = 13,350; HDL cholesterol: *n* = 12,993; LDL cholesterol: *n* = 12,963; triglycerides: *n* = 13,302) over an average of 3.7 y of follow-up. Mean ± SD changes presented in the top heading represent the average change in the markers over the follow-up. Forest plots represent linear regression coefficients with their 95% CIs adjusted for sociodemographic (age, sex, education, socioeconomic status), lifestyle (physical activity, smoking status), clinical (medication use, BMI), and dietary factors (total energy intake, food groups). *P < 0.05, **P < 0.01, ***P < 0.001. HDL-C, HDL cholesterol; LDL-C, LDL cholesterol.

**FIGURE 3:**
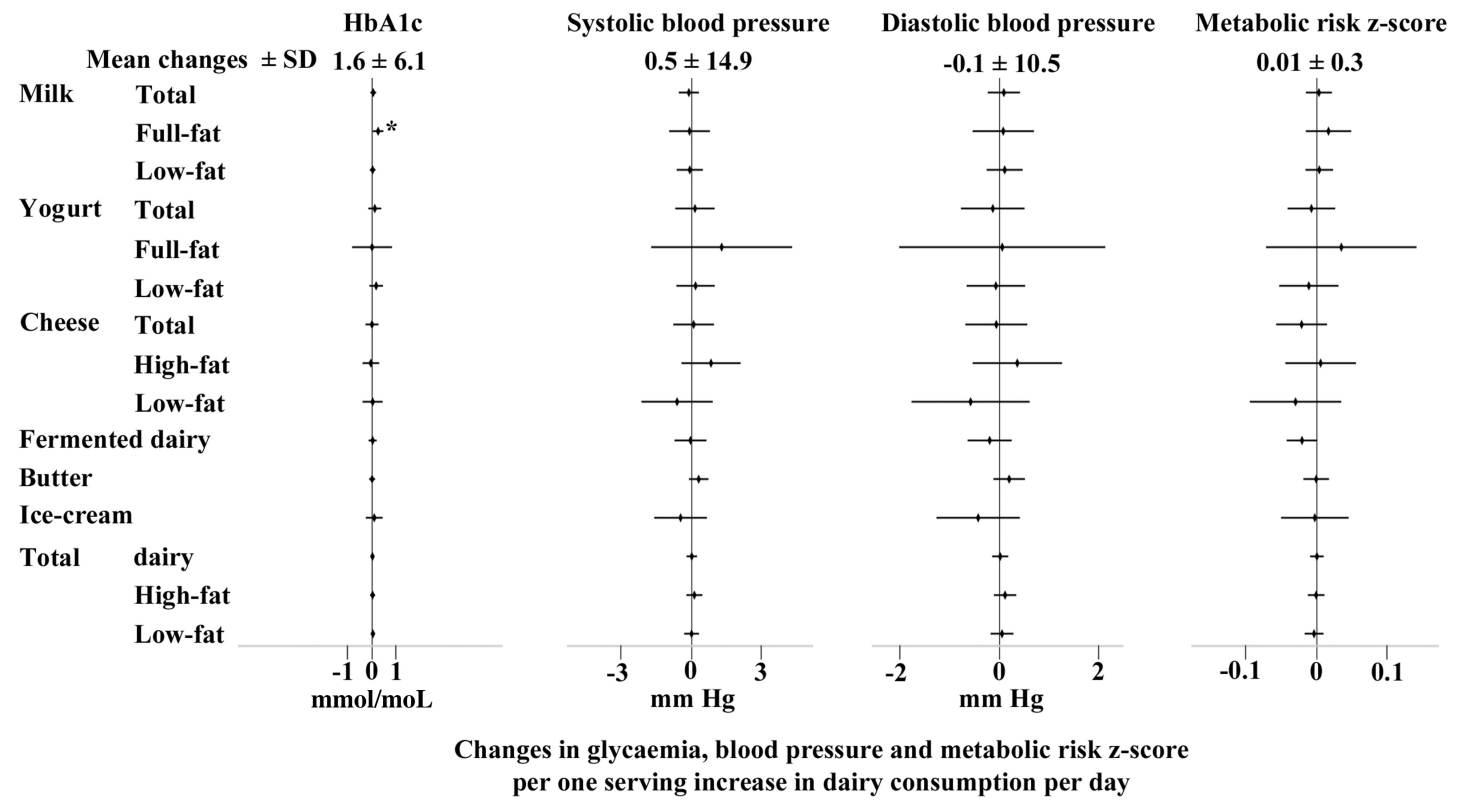
Associations of increases in dairy consumption (by 1 serving/d) with changes in glycemia (HbA1c: *n* = 6224), blood pressure (systolic blood pressure: *n* = 14,210; diastolic blood pressure: *n* = 14,231), and the metabolic-risk *z* score (*n* = 6033) over an average of 3.7 y of follow-up. Mean ± SD changes presented in the top heading represent the average change in the markers over the follow-up. Forest plots represent linear regression coefficients with their 95% CIs adjusted for sociodemographic (age, sex, education, socioeconomic status), lifestyle (physical activity, smoking status), clinical (medication use, BMI), and dietary factors (total energy intake, food groups). *P < 0.05, **P < 0.01, ***P < 0.001. HbA1c, glycated hemoglobin; HDL-C, HDL cholesterol; LDL-C, LDL cholesterol.

An increase in butter consumption by 1 serving/d was positively associated with an increase in LDL cholesterol in the positive control analysis (0.05 mmol/L; 95% CI: 0.02, 0.07 mmol/L) and also with an increase in total cholesterol (**Figure 2**). Changes in total cheese consumption were not associated with changes in lipid markers, but an increase in high-fat cheese consumption by 1 serving/d was associated with greater increases in each of total cholesterol by 0.12 mmol/L (95% CI: 0.04, 0.21 mmol/L), HDL cholesterol by 0.04 mmol/L (95% CI: 0.01, 0.07 mmol/L), and LDL cholesterol by 0.09 mmol/L (95% CI: 0.02, 0.16 mmol/L). Similar to butter, an increase in total high-fat dairy consumption was positively associated with a change in LDL cholesterol (0.04 mmol/L; 95% CI: 0.02, 0.06). Total and low-fat milk consumption was inversely associated with an increase in LDL cholesterol [−0.04 mmol/L (95% CI: −0.07, −0.01 mmol/L) and −0.03 mmol/L (95% CI: −0.06, −0.01 mmol/L) respectively]. An increase in habitual total and low-fat yogurt consumption was associated with a lower increase in total cholesterol by 0.06 mmol/L (95% CI: −0.12, −0.01 mmol/L) and in HDL cholesterol by 0.02 mmol/L (95% CI: −0.04, −0.01 mmol/L). An increase of 1 serving/d in total low-fat dairy consumption was associated with a lower increase in total and LDL cholesterol by 0.03 mmol/L (95% CI: −0.05, −0.01 mmol/L). The other associations with lipid markers were not significant for each of full-fat milk, yogurt, total or low-fat cheese, and fermented and total dairy products. Among nonlipid metabolic markers, a positive association was observed between an increase in full-fat milk and an increase in HbA1c (0.52 mmol/mol; 95% CI: 0.06, 0.97 mmol/mol) (**Figure 3**).

### Secondary analyses

We observed a significant interaction by age (*P*-interaction < 0.05): increases in high-fat cheese were positively associated with increases in body weight and BMI [0.87 kg (95% CI: 0.33, 1.41 kg) and 0.28 kg/m^2^(95% CI: 0.08, 0.48 kg/m^2^), respectively] among participants aged 50–60 y, but not among younger or older participants (*P*-interaction by age = 0.037 and 0.027, respectively; **Supplemental Table 4**). There were also significant interactions by sex. An increase in low-fat milk was inversely associated with an increase in the ratio of total to HDL cholesterol only among men but not women: −0.06 (95% CI: −0.1, −0.02) and 0.02 (95% CI: −0.02, 0.05), respectively (*P*-interaction = 0.02). An increase in cheese consumption was inversely associated with an increase in waist circumference only among women but not men: −0.48 cm (95% CI: −0.88, −0.08 cm) and 0.21 cm (95% CI: −0.32, 0.75 cm), respectively (*P*-interaction = 0.009). Other interactions or stratified associations were not significant. Results from the specified sensitivity analyses for different number of imputed datasets used, prevalent diseases, and possible attrition bias were not substantially different from results of the primary analysis (results not shown).

In the longitudinal analyses relating repeated measures of dairy consumption to repeated measures of cardiometabolic markers, butter intake was positively associated with LDL cholesterol (0.32 mmol/L; 95% CI: 0.15, 0.49 mmol/L; positive control; **Supplemental Table 5**). Some associations with markers of adiposity differed between the parallel change (primary) analyses and those from the secondary longitudinal analysis with repeated measures. For example, yogurt consumption was positively associated with body weight (0.32 kg; 95% CI: 0.15, 0.49 kg), BMI (0.49 kg/m^2^; 95% CI: 0.27, 0.17), and waist circumference (0.54 cm; 95% CI: 0.28, 0.79 cm) in secondary longitudinal analyses (**Supplemental Table 6**).

## Discussion

This large prospective study investigating changes in dairy consumption with parallel changes in cardiometabolic risk markers found diverse associations for different dairy subtypes. Our key findings were that *1*) increasing consumption of fermented dairy products (total yogurt, low-fat yogurt, and low-fat cheese) was associated with lower increases in body weight and BMI, *2*) increasing total high-fat dairy consumption was associated with higher increases in adiposity measures and major lipid markers, and *3*) increasing consumption of total low-fat dairy products was associated with lower increases in both total and LDL cholesterol.

### Findings in the context of other evidence

In pooled analyses of 3 cohorts, an increase in yogurt consumption was inversely associated with an increase in body weight (10), in agreement with our finding. Hypothesized mechanisms include that gut microbiota may reduce caloric effects of different macronutrients and/or influence appetite-related gastrointestinal hormones toward the direction of lowering body adiposity (29). Previous reports for cheese were inconsistent, indicating inverse or positive associations with changes in body weight (12). Our nonsignificant findings for other dairy subtypes and adiposity measures were also consistent with those from previous studies (12, 30–35), thereby increasing the specificity of the findings to potential biological effects of fermented dairy. In our secondary analysis we noted that some of the findings were sensitive to different statistical approaches. This difference highlights the importance and relevance of the study design and the analytical approach to the study findings. We noted that a prior observational study that examined associations between several dietary factors and weight change showed that results from parallel-change analyses were more consistent with evidence from randomized controlled trials than results from other approaches that used the baseline diet or changes in diet prior to change in body weight (11).

We found a positive association between increases in high-fat dairy consumption, including full-fat milk, and increases in body weight or BMI. These associations were heterogeneous by age and sex: for instance, more significant associations were observed among women and adults aged 50–60 y. Randomized controlled trials have suggested that total dairy consumption may increase body lean mass but decrease body fat mass, resulting in no significant change in body weight, with substantial heterogeneity in those effects (6). Available evidence on the potential heterogeneous effect of dairy products highlights the need for further research on different anthropometric compartments in diverse populations (36).

The positive association of total high-fat dairy products and butter with lipids as reported in our study and in randomized controlled trials might reflect the lipid-elevating effect of saturated fat, when it substitutes carbohydrates (37) or MUFAs or PUFAs (38). The association we observed for high-fat dairy products was mainly attributed to butter and high-fat cheese rather than full-fat milk. An increase in high-fat cheese consumption was also associated with higher increases in LDL cholesterol and the magnitude of the association was comparable with that for butter. Randomized controlled trials have shown that cheese leads to smaller increases in lipids compared with butter when fixing the ratio of saturated to polyunsaturated fat across the trial arms (39). This observation might be explained by the higher content of the milk-fat globule membrane in cheese than in butter (due to churning), which may reduce cholesterol absorption (40). This potential mechanism warrants further investigation in the context of habitual consumption and specific types of cheese, because high-fat cheese and butter could elevate LDL cholesterol in a general population, as our results indicated. Nevertheless, we should note that the saturated fat profile differs among different foods and null or inverse associations have been reported between saturated fat from dairy products and cardiometabolic disease outcomes (41). An explanation for the apparent discrepancy between the positive associations of high-fat dairy products with circulating lipids that we observed and the null or inverse associations of dairy fat with cardiometabolic disease outcomes in other research may be that there are multiple pathways linking dairy fat to cardiometabolic disease, and lipids, although important, constitute only 1 pathway (41). We also observed that an increase in low-fat dairy consumption was associated with a decrease in total and LDL cholesterol. This finding is biologically plausible because dairy calcium intake may improve lipid profiles by inhibiting cholesterol reabsorption, inhibiting lipogenesis, or increasing lipolysis (42, 43), whereas low-fat dairy does not exert the cholesterol-raising effect of saturated fat. However, the finding was at variance with evidence from prior trials (44) and observational studies (10). The inconsistency may be due to inconsistent control arms and short durations in trials to detect the effect we estimated and different analytical approaches undertaken in observational studies (11).

Randomized controlled trials on the effects of dairy consumption on glycemic traits have been primarily limited to interventions with a mixture of dairy types and have showed null associations between dairy consumption and glycemia or blood pressure (44), in agreement with our results. Limited evidence also shows null effects of milk on glycemia (45, 46). We observed that increased habitual full-fat milk consumption was positively associated with increased HbA1c concentrations, but the reasons for this finding are unclear.

### Strengths and limitations

Strengths of this study include the large sample size and the availability of repeated measures of dairy consumption and cardiometabolic markers at the same time points. This enabled us to investigate associations of changes in the dietary exposure with parallel changes in outcomes, a recommended approach (11), although not adopted by many previous studies (10). Limitations of our work include residual confounding and bias due to the observational design of our study, thereby limiting causal inference. We cannot exclude the possibility of misreporting due to the use of the self-reported FFQ, which might have caused measurement error in changes in dietary consumption and covariates and the assessment of the dairy subtypes and the nutritional content (e.g., fat and its types) of the different types of cheese, for example. Such error could not be quantified, possibly introducing an unknown direction of bias. Due to the lack of repeated measures for biomarkers in our study we could not assess associations with changes in other lipid markers such as apolipoproteins, which have been shown to be important indicators of the association of saturated fat with cardiovascular risk (47). While we assessed statistical significance of the associations of interest, it remains challenging to assess clinical significance of the findings from this single study and based on single dietary items as they are consumed within overall dietary patterns.

## Conclusions

Using a prospective analysis of parallel changes in dairy consumption and markers of metabolic risk, we found differing associations of different dairy subtypes with adiposity and lipidemia. Our finding of an inverse association of an increase in the consumption of fermented dairy products, yogurt (total and low-fat), or low-fat cheese with an increase in body weight provides a potential pathway for the previously described inverse association of fermented dairy consumption with type 2 diabetes (19). These findings contribute to greater understanding on differing associations of subtypes of dairy products with cardiometabolic health. Rather than analyzing fermented and nonfermented dairy together, further work specifically on yogurt or yogurt plus cheese is warranted for future clinical and observational research and for dietary modification targeting weight management.

## Supplementary Material

nqz335_Supplemental_File

## Notes

The EPIC-Norfolk study (DOI 10.22025/2019.10.105.00004) has received funding from the Medical Research Council (MR/N003284/1 and MC-UU_12015/1) and Cancer Research UK (C864/A14136). This work was supported by the Medical Research Council Epidemiology Unit core funding (MC_UU_12015/1 and MC_UU_12015/5). NJW and NGF acknowledge support from the National Institute for Health Research (NIHR) Cambridge Biomedical Research Centre (IS-BRC-1215-20014), and NJW is an NIHR Senior Investigator. ET held a PhD scholarship from the Cambridge Trust and the Medical Research Council Epidemiology Unit. The funders were not involved in the design, implementation, analysis, or interpretation of the data.

Supplemental Tables 1–6 and Supplemental Figure 1 are available from the “Supplementary data” link in the online posting of the article and from the same link in the online table of contents at https://academic.oup.com/ajcn/.

FI and NGF contributed equally to this work.

Requests for accessing data described in the manuscript should be made to the EPIC-Norfolk Management Committee (e-mail: datasharing@mrc-epid.cam.ac.uk)

Abbreviations used: EPAQ2, EPIC Physical Activity Questionnaire 2; EPIC, European Investigation into Cancer and Nutrition; FFQ, food-frequency questionnaire; HbA1c, glycated hemoglobin.
