# Supplementary material for: The associations of longitudinal changes in consumption of total and types of dairy products and markers of metabolic risk and adiposity: findings from the European Investigation into Cancer and Nutrition (EPIC)–Norfolk study, United Kingdom
Source: Am J Clin Nutr. 2020 Jan 8;111(5):1018–26. doi: 10.1093/ajcn/nqz335 (PMC7198306; doi:10.1093/ajcn/nqz335)
Supplement: nqz335_Supplemental_File [file nqz335_supplemental_file.docx]

**The associations of longitudinal changes in consumption of total and types of dairy products and markers of metabolic risk and adiposity: findings from the EPIC-Norfolk study, United Kingdom, Trichia et al.**

**Online Supplementary Material**

Contents

[**Supplementary Figure 1**. Participants selection for the analyses of associations of dairy consumption with cardio-metabolic markers in over 15,000 adults of the EPIC Norfolk Study, UK. ^1^Outliers of total energy intake: <800 and >4,000 kcal/d for men and <500 and >3,500 kcal/d for women, ^2^Numbers slightly varied depending on missing information of each outcome and exclusion of outliers of the change in the outcomes based on the bottom and top percentiles (See Supplemental Table 4). EPIC: European Prospective Investigation into Cancer and Nutrition; UK: United Kingdom. 2](#_Toc25067437)

[**Supplementary Table 1**. Descriptive characteristics1 of markers of metabolic risk and adiposity at baseline, first follow-up, second follow-up and the change between baseline and first follow-up in the EPIC-Norfolk study, UK 3](#_Toc25067438)

[**Supplementary Table 2**. Descriptive characteristics of socio-demographic, behavioural, clinical and non-dairy dietary factors at baseline, first follow-up and the change between baseline and first follow-up in the EPIC-Norfolk study, UK^1^ 4](#_Toc25067439)

[**Supplementary Table 3**. Associations of the change in total and types of dairy products with the change in markers of adiposity, lipid markers, haemoglobin A1c, blood pressure measures, and metabolic risk z-score from baseline to the first follow-up after a mean of 3.7 years in the EPIC-Norfolk study, UK 6](#_Toc25067440)

[**Supplementary Table 4**. Stratified associations of the change in total and types of dairy products with the change in cardio-metabolic markers m baseline to the first follow-up: analysis stratified by significant effect modifiers in the EPIC-Norfolk study, UK^1^ 15](#_Toc25067441)

[**Supplementary Table 5**. Associations of the repeated measures of total and types of dairy products at baseline (1993-1997) and first follow-up (1998-2000) with the repeated measures of lipid markers at the first and the second (2004-2011) follow-up in the EPIC-Norfolk study, UK^1^ 17](#_Toc25067442)

[**Supplementary Table 6**. Longitudinal associations of the repeated measures of total and types of dairy products at baseline (1993-1997) and first follow-up (1998-2000) with the repeated measures of the markers of body weight and composition at the first and the second (2004-2011) follow-up in the EPIC-Norfolk study, UK^1^ 18](#_Toc25067443)


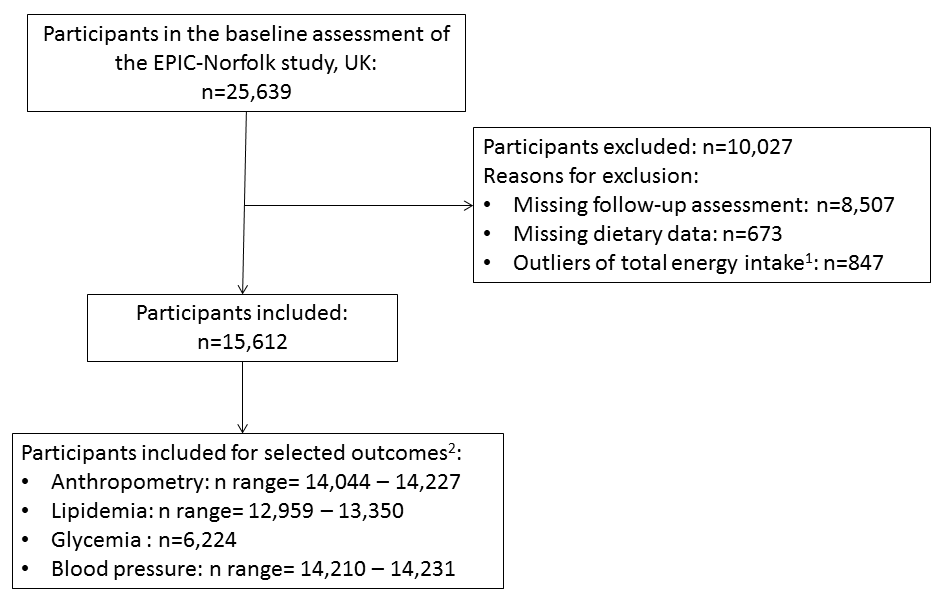


# **Supplementary Figure 1**. Participants selection for the analyses of associations of dairy consumption with cardio-metabolic markers in over 15,000 adults of the EPIC Norfolk Study, UK. ^1^Outliers of total energy intake: <800 and >4,000 kcal/d for men and <500 and >3,500 kcal/d for women, ^2^Numbers slightly varied depending on missing information of each outcome and exclusion of outliers of the change in the outcomes based on the bottom and top percentiles (See Supplemental Table 4). EPIC: European Prospective Investigation into Cancer and Nutrition; UK: United Kingdom.

| **Supplementary Table 1**. Descriptive characteristics1 of markers of metabolic risk and adiposity at baseline, first follow-up, second follow-up and the change between baseline and first follow-up in the EPIC-Norfolk study, UK | | | |
| --- | --- | --- | --- |
|  | **Baseline** | **1st follow-up** | **Change** |
| **Anthropometric markers** |  |  |  |
| Weight (kg) | 73.0 ± 12.8 | 74.1± 13.2 | 1.3 ± 4.0 |
| BMI (kg/m^2^) | 26.1 ± 3.7 | 26.7 ± 4.0 | 0.6 ± 1.4 |
| Waist circumference (cm) | 87.3 ± 12.1 | 88.0 ± 12.5 | 0.8 ± 5.5 |
| Waist-to-hip ratio | 0.8 ± 0.1 | 0.8 ± 0.1 | 0.0 ± 0.0 |
| **Lipid markers** |  |  |  |
| Total / HDL cholesterol | 4.7 ± 1.6 | 4.4 ± 1.6 | -0.2 ± 1.2 |
| Total cholesterol (mmol/L) | 6.2 ± 1.2 | 6.1 ± 1.2 | -0.1 ± 1.0 |
| HDL cholesterol (mmol/L) | 1.4 ± 0.4 | 1.5 ± 0.5 | 0.1 ± 0.3 |
| LDL cholesterol (mmol/L) | 4.0 ± 1.0 | 3.8 ± 1.0 | -0.2 ± 0.9 |
| Triglycerides (mmol/L) | 1.8 ± 1.1 | 1.9 ± 1.1 | 0.1 ± 0.9 |
| **Other markers** |  |  |  |
| HbA1c (mmol/mol) | 34.9 ± 8.5 | 36.6 ± 7.3 | 1.6 ± 6.1 |
| Systolic blood pressure (mm Hg) | 134.3 ± 17.9 | 135.1 ± 18.2 | 0.5 ± 14.9 |
| Diastolic blood pressure (mmHg) | 82.0 ± 11.0 | 81.9 ± 11.2 | -0.1 ± 10.5 |
| Metabolic risk z-score | -0.02 ± 0.6 | 0.0 ±0.6 | 0.01 ± 0.3 |
| ^1^Descriptive statistics are presented as Mean ± SD, n=15,612  EPIC: European Prospective Investigation into Cancer and Nutrition; HbA1c: Hemoglobin A1c; UK: United Kingdom | | | |

| **Supplementary Table 2**. Descriptive characteristics of socio-demographic, behavioural, clinical and non-dairy dietary factors at baseline, first follow-up and the change between baseline and first follow-up in the EPIC-Norfolk study, UK^1^ | | | |
| --- | --- | --- | --- |
|  | **Baseline** | **1st follow-up** | **Change** |
| **Socio-demographic factors** |  |  |  |
| Age (years) | 58.6 ± 8.9 | 62.1 ± 9.0 | 3.2 ± 0.8 |
| Sex^2^, women | 56.2 | 56.2 |  |
| Educational level^2^, medium | 41.9 | 41.9 |  |
| high | 14.7 | 14.6 |  |
| Age completing education (years)^3^ | 8.7 ± 12.2 | 8.7 ± 12.2 | 0.0 ± 0.0 |
| Socio-economic status^2^, medium | 16.7 | 16.7 |  |
| high | 46.7 | 46.7 |  |
| Marital status^2,3^, married | 82.6 | 82.6 |  |
| widowed / separated | 13.4 | 13.5 |  |
| **Lifestyle factors** |  |  |  |
| Smoking status^2^, former smoker | 41.4 | 42.7 |  |
| current smoker | 9.4 | 8.1 |  |
| Physical activity^2,4^, moderately inactive | 29.6 | 15.9 |  |
| moderately active | 24.3 | 20.3 |  |
| active | 19.3 | 15.3 |  |
| Energy intake (kJ/d)^4^ | 2,018 ± 552 | 1,929 ± 529 | -88.6 ± 478.5 |
| **Medications / Supplements** |  |  |  |
| Lipid-lowering medication^2^ | 1.5 | 4.7 |  |
| Anti-hypertensive medication^2^ | 16.4 | 21.8 |  |
| Hormonal therapy^2^ | 12.3 | 12.5 |  |
| Dietary supplements^2,4^ | 49.6 | 55.5 |  |
| **Non-dairy food dietary factors (g/d)^4^** |  |  |  |
| Fruits | 250.2 ± 180.4 | 263.6 ± 187.7 | 12.2 ± 170.9 |
| Vegetables | 241.8 ± 124.0 | 240.8 ± 123.3 | -2.1 ± 110.8 |
| Potatoes | 115.2 ± 60.5 | 111.7 ± 59.5 | -3.7 ± 69.3 |
| Legumes | 60.0 ± 37.6 | 56.0 ± 35.8 | -3.9 ± 38.3 |
| Processed cereals | 82.1 ± 54.1 | 78.9 ± 49.6 | -2.9 ± 53.0 |
| Whole-grain cereals | 78.9 ± 78.0 | 69.1 ± 73.1 | -10.3 ± 75.1 |
| Poultry and eggs | 37.9 ± 23.9 | 37.5 ± 24.7 | -0.3 ± 25.9 |
| Red meat | 62.0 ± 40.5 | 57.7 ± 39.5 | -4.1 ± 42.4 |
| Processed meat | 27.9 ± 22.9 | 27.5 ± 22.3 | -0.4 ± 21.8 |
| Fish | 37.7 ± 25.8 | 37.4 ± 25.5 | -0.2 ± 25.4 |
| Sauces | 19.5 ± 17.9 | 19.3 ± 18.6 | -0.2 ± 20.4 |
| Margarine | 16.6 ± 16.3 | 14.4 ± 15.1 | -2.2 ± 16.6 |
| Nuts | 2.5 ± 7.4 | 2.5 ± 7.7 | 0.0 ± 9.2 |
| Sweet snacks | 116.6 ± 84.6 | 108.3 ± 82.3 | -8.6 ± 72.2 |
| Sugar-sweetened beverages | 33.1 ± 72.1 | 33.7 ± 75.6 | 0.9 ± 83.2 |
| Artificially sweetened beverages | 36.9 ± 104.9 | 36.0 ± 104.3 | -0.3 ± 101.1 |
| Fruit juice | 50.9 ± 69.1 | 55.1 ± 72.0 | 4.0 ± 73.7 |
| Regular coffee | 329.5 ± 320.6 | 298.6 ± 301.2 | -27.9 ± 252.8 |
| Decaffeinated coffee | 87.6 ± 207.1 | 76.8 ± 191.7 | -10.2 ± 187.7 |
| Tea | 632.0 ± 365.2 | 617.1 ± 360.5 | -18.2 ± 250.6 |
| Alcoholic beverages | 128.8 ± 232.2 | 125.4 ± 221.9 | -1.5 ± 154.7 |
| ^1^Continuous variables are presented as mean ± SD and categorical variables are presented as column percentages, n=15,612  ^2^Reference categories: sex: men; educational level: low; socio-economic status: low; physical activity: inactive; lipid-lowering medication: no; anti-hypertensive medication: no; hormonal therapy: no; dietary supplements: no  ^3^Missing values <5% at baseline and follow-up  ^4^Missing values <5% at baseline, but 20-50% at follow-up  EPIC: European Prospective Investigation into Cancer and Nutrition; UK: United Kingdom | | | |

| **Supplementary Table 3**. Associations of the change in total and types of dairy products with the change in markers of adiposity, lipid markers, haemoglobin A1c, blood pressure measures, and metabolic risk z-score from baseline to the first follow-up after a mean of 3.7 years in the EPIC-Norfolk study, UK | | | | |
| --- | --- | --- | --- | --- |
|  | **Weight (kg)** | **BMI (kg/m**^2^) | **Waist (cm)** | **Waist / Hip circumference** |
| **Mean ± SD of change** | 1.3 ± 4.0 | 0.6 ± 1.4 | 0.8 ± 5.5 | -0.001 ± 0.046 |
| **Participants (n)** | 14,044 | 14,134 | 14,227 | 14,213 |
| **Dairy consumption (servings/d)^1^** | b (95% CI) | b (95% CI) | b (95% CI) | b (95% CI) |
| **Milk** |  |  |  |  |
| Demographic, lifestyle, energy^2^ | 0.05(-0.06, 0.16) | 0.02(-0.02, 0.06) | -0.03(-0.18, 0.13) | -0.001(-0.002, 0.001) |
| + Diet^3^ | 0.05(-0.06, 0.17) | 0.02(-0.02, 0.06) | -0.03(-0.20, 0.13) | -0.001(-0.002, 0.001) |
| + BMI^4^ |  |  | -0.07(-0.19, 0.06) | -0.001(-0.002, 0.001) |
| **Full-fat milk** |  |  |  |  |
| Demographic, lifestyle, energy^2^ | 0.25(0.03, 0.46) | 0.09(0.01, 0.18) | 0.27(-0.02, 0.57) | 0.000(-0.002, 0.002) |
| + Diet^3^ | 0.23(0.02, 0.45) | 0.09(0.01, 0.17) | 0.25(-0.04, 0.55) | 0.000(-0.002, 0.002) |
| + BMI^4^ |  |  | 0.04(-0.19, 0.28) | -0.001(-0.003, 0.002) |
| **Low-fat milk** |  |  |  |  |
| Demographic, lifestyle, energy^2^ | 0.05(-0.06, 0.15) | 0.02(-0.02, 0.06) | 0.01(-0.15, 0.17) | 0.000(-0.002, 0.001) |
| + Diet^3^ | 0.05(-0.06, 0.16) | 0.02(-0.02, 0.06) | -0.01(-0.16, 0.15) | 0.000(-0.002, 0.001) |
| + BMI^4^ |  |  | -0.04(-0.17, 0.08) | -0.001(-0.002, 0.001) |
| **Yogurt** |  |  |  |  |
| Demographic, lifestyle, energy^2^ | -0.27(-0.49, -0.05) | -0.11(-0.19, -0.02) | -0.41(-0.71, -0.10) | -0.002(-0.004, 0.001) |
| + Diet^3^ | -0.23(-0.46, -0.01) | -0.09(-0.18, 0.00) | -0.38(-0.68, -0.07) | -0.002(-0.004, 0.001) |
| + BMI^4^ |  |  | -0.13(-0.41, 0.14) | -0.001(-0.003, 0.002) |
| **Full-fat yogurt** |  |  |  |  |
| Demographic, lifestyle, energy^2^ | -0.07(-0.90, 0.75) | -0.03(-0.31, 0.26) | 0.02(-1.06, 1.10) | 0.002(-0.010, 0.010) |
| + Diet^3^ | -0.04(-0.86, 0.78) | -0.01(-0.30, 0.27) | 0.02(-1.06, 1.10) | 0.002(-0.010, 0.010) |
| + BMI^4^ |  |  | 0.04(-0.93, 1.02) | 0.002(-0.010, 0.010) |
| **Low-fat yogurt** |  |  |  |  |
| Demographic, lifestyle, energy^2^ | -0.34(-0.59, -0.10) | -0.14(-0.23, -0.05) | -0.44(-0.79, -0.09) | -0.002(-0.010, 0.001) |
| + Diet^3^ | -0.31(-0.56, -0.06) | -0.13(-0.22, -0.04) | -0.41(-0.77, -0.05) | -0.002(-0.005, 0.001) |
| + BMI^4^ |  |  | -0.10(-0.38, 0.19) | -0.001(-0.004, 0.002) |
| **Cheese** |  |  |  |  |
| Demographic, lifestyle, energy^2^ | -0.18(-0.44, 0.08) | -0.07(-0.17, 0.03) | -0.36(-0.72, -0.01) | -0.002(-0.005, 0.000) |
| + Diet^3^ | -0.14(-0.39, 0.12) | -0.06(-0.15, 0.04) | -0.34(-0.70, 0.02) | -0.002(-0.005, 0.000) |
| + BMI^4^ |  |  | -0.19(-0.52, 0.14) | -0.002(-0.005, 0.001) |
| **High-fat cheese** |  |  |  |  |
| Demographic, lifestyle, energy^2^ | 0.51(0.16, 0.86) | 0.18(0.06, 0.30) | 0.48(-0.06, 1.01) | 0.002(-0.003, 0.010) |
| + Diet^3^ | 0.48(0.13, 0.84) | 0.17(0.05, 0.29) | 0.41(-0.11, 0.94) | 0.001(-0.003, 0.010) |
| + BMI^4^ |  |  | -0.01(-0.44, 0.42) | 0.000(-0.004, 0.004) |
| **Low-fat cheese** |  |  |  |  |
| Demographic, lifestyle, energy^2^ | -0.73(-1.07, -0.40) | -0.27(-0.39, -0.15) | -1.08(-1.57, -0.59) | -0.010(-0.010, -0.001) |
| + Diet^3^ | -0.64(-0.97, -0.31) | -0.23(-0.36, -0.11) | -1.01(-1.50, -0.51) | -0.010(-0.010, -0.001) |
| + BMI^4^ |  |  | -0.36(-0.82, 0.10) | -0.003(-0.010, 0.001) |
| **Fermented dairy products** |  |  |  |  |
| Demographic, lifestyle, energy^2^ | -0.23(-0.37, -0.08) | -0.09(-0.14, -0.03) | -0.31(-0.58, -0.04) | -0.002(-0.004, 0.000) |
| + Diet^3^ | -0.20(-0.34, -0.06) | -0.07(-0.13, -0.02) | -0.30(-0.56, -0.04) | -0.002(-0.004, 0.000) |
| + BMI^4^ |  |  | -0.10(-0.30, 0.11) | -0.001(-0.003, 0.001) |
| **Butter** |  |  |  |  |
| Demographic, lifestyle, energy^2^ | 0.08(-0.03, 0.18) | 0.02(-0.02, 0.06) | 0.16(0.002, 0.32) | 0.001(0.000, 0.002) |
| + Diet^3^ | 0.08(-0.03, 0.19) | 0.02(-0.02, 0.06) | 0.16(-0.01, 0.33) | 0.001(0.000, 0.002) |
| + BMI^4^ |  |  | 0.10(-0.03, 0.24) | 0.001(0.000, 0.002) |
| **Ice-cream** |  |  |  |  |
| Demographic, lifestyle, energy^2^ | 0.24(-0.07, 0.56) | 0.08(-0.04, 0.19) | 0.20(-0.35, 0.74) | 0.000(-0.004, 0.004) |
| + Diet^3^ | 0.23(-0.08, 0.55) | 0.07(-0.04, 0.19) | 0.16(-0.37, 0.70) | 0.000(-0.004, 0.004) |
| + BMI^4^ |  |  | 0.02(-0.44, 0.47) | -0.001(-0.004, 0.003) |
| **Total dairy products** |  |  |  |  |
| Demographic, lifestyle, energy^2^ | 0.01(-0.06, 0.07) | 0.00(-0.02, 0.03) | -0.01(-0.10, 0.09) | 0.000(-0.001, 0.000) |
| + Diet^3^ | 0.02(-0.05, 0.09) | 0.00(-0.02, 0.03) | -0.01(-0.12, 0.09) | 0.000(-0.001, 0.001) |
| + BMI^4^ |  |  | -0.001(-0.08, 0.08) | 0.000(-0.001, 0.001) |
| **High-fat dairy products** |  |  |  |  |
| Demographic, lifestyle, energy^2^ | 0.13(0.05, 0.21) | 0.04(0.02, 0.07) | 0.12(0.01, 0.24) | 0.000(-0.001, 0.001) |
| + Diet^3^ | 0.13(0.05, 0.21) | 0.04(0.01, 0.07) | 0.11(-0.01, 0.22) | 0.000(-0.001, 0.001) |
| + BMI^4^ |  |  | 0.02(-0.09, 0.13) | 0.000(-0.001, 0.001) |
| **Low-fat dairy products** |  |  |  |  |
| Demographic, lifestyle, energy^2^ | -0.08(-0.16, -0.01) | -0.03(-0.06, -0.002) | -0.15(-0.28, -0.02) | -0.001(-0.002, 0.000) |
| + Diet^3^ | -0.07(-0.14, 0.01) | -0.02(-0.05, 0.004) | -0.15(-0.29, -0.02) | -0.001(-0.002, 0.000) |
| + BMI^4^ |  |  | -0.07(-0.20, 0.05) | -0.001(-0.002, 0.000) |

‡ Servings as defined by Food Standards Agency 2002: Milk- 1 average glass (200g); Yogurt- 125g carton; Cheese- medium serving (40g); Single cream- 1 tablespoon (15g); Double cream- 1 tablespoon (30g); Butter- 1 teaspoon (10g); Ice-cream- 1 average scoop/tub (60g)

§ Linear regression model 1: age (years), sex, educational level (low, medium, high), age at completion of full-time education (years), marital status (single, married, widowed or separated), socio-economic status based on occupation (low: technical/semi-routine and routine occupations medium: lower managerial / intermediate occupations; high: professional / higher managerial occupations), individual follow-up time (years), physical activity level (inactive, moderately inactive, moderately active, active), smoking status (never, former and current smoker), lipid-lowering medication (Yes, No), anti-hypertensive medication (Yes, No), hormone-replacement therapy (Yes, No, Men), total energy intake (kcal/day)

ǁ Linear regression model 2: Model 1 + intakes (g/d) of fruit, vegetables, potatoes, legumes, nuts, processed cereals, whole-grain cereals, poultry and eggs, red meat, processed meat, fish, sauces, margarine, sweet snacks, sugar-sweetened beverages, artificially sweetened beverages, fruit juice, coffee, tea and alcoholic beverages and dietary supplement use (Yes, No)

# Linear regression model 3: Model 2 + BMI (kg/m2)

EPIC: European Prospective Investigation into Cancer and Nutrition; UK: United Kingdom

| **Supplementary** T**able 3**. (continued on circulating lipids) | | | | | |
| --- | --- | --- | --- | --- | --- |
|  | **Total / HDL cholesterol †** | **Total cholesterol (mmol/L)** | **HDL cholesterol (mmol/L)** | **LDL cholesterol (mmol/L)** | **Triglycerides (mmol/L)** |
| **Mean ±** **SD of change** | -0.2 ± 1.2 | -0.1 ± 1.0 | 0.1 ± 0.3 | -0.2 ± 0.9 | 0.1 ± 0.9 |
| **Participants (n)** | 12,959 | 13,350 | 12,993 | 12,963 | 13,302 |
| **Dairy consumption (servings/d)^1^** | b (95% CI) | b (95% CI) | b (95% CI) | b (95% CI) | b (95% CI) |
| **Milk** |  |  |  |  |  |
| Demographic, lifestyle, energy^2^ | -0.02(-0.05, 0.01) | -0.03(-0.06, 0.01) | 0.002(-0.01, 0.01) | -0.04(-0.06, -0.01) | 0.02(-0.01, 0.05) |
| + Diet^3^ | -0.02(-0.05, 0.02) | -0.03(-0.06, 0.01) | 0.003(-0.01, 0.01) | -0.04(-0.07, -0.01) | 0.02(-0.01, 0.05) |
| + BMI^4^ | -0.02(-0.05, 0.01) | -0.03(-0.06, 0.01) | 0.003(-0.01, 0.01) | -0.04(-0.07, -0.01) | 0.02(-0.01, 0.04) |
| **Full-fat milk** |  |  |  |  |  |
| Demographic, lifestyle, energy^2^ | 0.03(-0.02, 0.09) | 0.03(-0.02, 0.08) | 0.00(-0.02, 0.02) | 0.04(-0.004, 0.09) | 0.002(-0.04, 0.04) |
| + Diet^3^ | 0.03(-0.02, 0.09) | 0.03(-0.02, 0.08) | 0.00(-0.02, 0.02) | 0.04(-0.005, 0.09) | -0.001(-0.04, 0.04) |
| + BMI^4^ | 0.02(-0.04, 0.07) | 0.02(-0.03, 0.07) | 0.002(-0.01, 0.02) | 0.03(-0.01, 0.08) | -0.01(-0.06, 0.03) |
| **Low-fat milk** |  |  |  |  |  |
| Demographic, lifestyle, energy^2^ | -0.02(-0.05, 0.01) | -0.02(-0.05, 0.02) | 0.001(-0.01, 0.01) | -0.03(-0.06, -0.01) | 0.02(-0.001, 0.05) |
| + Diet^3^ | -0.01(-0.04, 0.01) | -0.01(-0.04, 0.02) | 0.001(-0.01, 0.01) | -0.03(-0.06, -0.01) | 0.03(0.001, 0.05) |
| + BMI^4^ | -0.02(-0.04, 0.01) | -0.02(-0.05, 0.01) | 0.002(-0.01, 0.01) | -0.03(-0.06, -0.01) | 0.02(0.00, 0.05) |
| **Yogurt** |  |  |  |  |  |
| Demographic, lifestyle, energy^2^ | -0.01(-0.07, 0.05) | -0.08(-0.14, -0.03) | -0.02(-0.04, -0.01) | -0.02(-0.07, 0.02) | -0.06(-0.12, -0.01) |
| + Diet^3^ | -0.01(-0.07, 0.05) | -0.08(-0.14, -0.02) | -0.02(-0.04, -0.004) | -0.02(-0.07, 0.03) | -0.06(-0.11, -0.01) |
| + BMI^4^ | 0.01(-0.05, 0.07) | -0.06(-0.12, -0.01) | -0.02(-0.04, -0.01) | -0.01(-0.06, 0.04) | -0.04(-0.09, 0.005) |
| **Full-fat yogurt** |  |  |  |  |  |
| Demographic, lifestyle, energy^2^ | 0.19(-0.03, 0.41) | 0.10(-0.08, 0.29) | -0.04(-0.11, 0.03) | 0.13(-0.05, 0.30) | -0.01(-0.18, 0.15) |
| + Diet^3^ | 0.18(-0.04, 0.40) | 0.08(-0.10, 0.27) | -0.04(-0.11, 0.03) | 0.11(-0.07, 0.28) | -0.01(-0.17, 0.16) |
| + BMI^4^ | 0.17(-0.05, 0.40) | 0.08(-0.10, 0.26) | -0.04(-0.11, 0.03) | 0.10(-0.07, 0.28) | -0.01(-0.17, 0.15) |
| **Low-fat yogurt** |  |  |  |  |  |
| Demographic, lifestyle, energy^2^ | -0.01(-0.08, 0.05) | -0.09(-0.14, -0.03) | -0.02(-0.04, 0.00) | -0.03(-0.08, 0.02) | -0.07(-0.12, -0.02) |
| + Diet^3^ | -0.02(-0.08, 0.05) | -0.08(-0.14, -0.02) | -0.02(-0.04, 0.002) | -0.03(-0.08, 0.03) | -0.07(-0.12, -0.01) |
| + BMI^4^ | 0.01(-0.05, 0.07) | -0.06(-0.12, -0.01) | -0.02(-0.04, 0.00) | -0.01(-0.06, 0.04) | -0.05(-0.10, 0.00) |
| **Cheese** |  |  |  |  |  |
| Demographic, lifestyle, energy^2^ | -0.02(-0.09, 0.05) | 0.04(-0.01, 0.10) | 0.02(-0.01, 0.04) | 0.02(-0.03, 0.08) | 0.01(-0.05, 0.06) |
| + Diet^3^ | -0.02(-0.09, 0.05) | 0.04(-0.01, 0.10) | 0.02(-0.004, 0.04) | 0.02(-0.03, 0.07) | 0.01(-0.04, 0.06) |
| + BMI^4^ | -0.01(-0.08, 0.05) | 0.05(-0.01, 0.10) | 0.02(-0.01, 0.04) | 0.02(-0.03, 0.08) | 0.02(-0.03, 0.07) |
| **High-fat cheese** |  |  |  |  |  |
| Demographic, lifestyle, energy^2^ | 0.03(-0.07, 0.13) | 0.16(0.06, 0.25) | 0.03(0.01, 0.06) | 0.11(0.04, 0.19) | 0.02(-0.05, 0.10) |
| + Diet^3^ | 0.02(-0.07, 0.12) | 0.15(0.06, 0.24) | 0.03(0.01, 0.06) | 0.11(0.04, 0.18) | 0.02(-0.06, 0.09) |
| + BMI^4^ | -0.01(-0.10, 0.08) | 0.12(0.04, 0.21) | 0.04(0.01, 0.07) | 0.09(0.02, 0.16) | -0.01(-0.09, 0.06) |
| **Low-fat cheese** |  |  |  |  |  |
| Demographic, lifestyle, energy^2^ | -0.08(-0.18, 0.02) | -0.07(-0.17, 0.03) | 0.001(-0.03, 0.03) | -0.05(-0.15, 0.05) | -0.03(-0.11, 0.04) |
| + Diet^3^ | -0.08(-0.18, 0.03) | -0.06(-0.16, 0.04) | 0.003(-0.03, 0.03) | -0.05(-0.15, 0.06) | -0.02(-0.10, 0.06) |
| + BMI^4^ | -0.04(-0.14, 0.06) | -0.03(-0.13, 0.07) | -0.003(-0.03, 0.03) | -0.03(-0.13, 0.08) | 0.02(-0.06, 0.09) |
| **Fermented dairy products** |  |  |  |  |  |
| Demographic, lifestyle, energy^2^ | -0.03(-0.07, 0.01) | -0.02(-0.06, 0.02) | 0.002(-0.01, 0.01) | -0.003(-0.04, 0.03) | -0.03(-0.07, 0.00) |
| + Diet^3^ | -0.03(-0.07, 0.01) | -0.02(-0.06, 0.02) | 0.004(-0.01, 0.02) | -0.004(-0.04, 0.03) | -0.03(-0.06, 0.002) |
| + BMI^4^ | -0.02(-0.06, 0.02) | -0.01(-0.04, 0.03) | 0.002(-0.01, 0.02) | 0.01(-0.03, 0.04) | -0.02(-0.05, 0.01) |
| **Butter** |  |  |  |  |  |
| Demographic, lifestyle, energy^2^ | 0.03(0.003, 0.07) | 0.06(0.04, 0.08) | 0.003(-0.01, 0.01) | 0.05(0.03, 0.07) | 0.004(-0.02, 0.03) |
| + Diet^3^ | 0.03(-0.01, 0.06) | 0.06(0.03, 0.08) | 0.004(-0.01, 0.02) | 0.05(0.02, 0.07) | 0.002(-0.03, 0.03) |
| + BMI^4^ | 0.02(-0.01, 0.06) | 0.05(0.03, 0.08) | 0.01(-0.01, 0.02) | 0.05(0.02, 0.07) | -0.001(-0.03, 0.03) |
| **Ice-cream** |  |  |  |  |  |
| Demographic, lifestyle, energy^2^ | 0.00(-0.08, 0.08) | 0.04(-0.03, 0.12) | -0.01(-0.03, 0.02) | -0.01(-0.07, 0.06) | 0.08(0.02, 0.15) |
| + Diet^3^ | 0.01(-0.07, 0.10) | 0.05(-0.03, 0.13) | -0.01(-0.03, 0.02) | 0.002(-0.06, 0.07) | 0.08(0.02, 0.15) |
| + BMI^4^ | 0.01(-0.08, 0.09) | 0.04(-0.03, 0.12) | 0.00(-0.03, 0.02) | -0.001(-0.07, 0.07) | 0.07(0.01, 0.14) |
| **Total dairy products** |  |  |  |  |  |
| Demographic, lifestyle, energy^2^ | 0.003(-0.02, 0.02) | 0.02(0.00, 0.04) | 0.002(-0.004, 0.01) | 0.01(-0.01, 0.02) | 0.01(-0.003, 0.03) |
| + Diet^3^ | 0.001(-0.02, 0.02) | 0.02(0.00, 0.03) | 0.002(-0.004, 0.01) | 0.01(-0.01, 0.02) | 0.01(-0.002, 0.03) |
| + BMI^4^ | 0.002(-0.02, 0.02) | 0.02(0.00, 0.04) | 0.003(-0.003, 0.01) | 0.01(-0.01, 0.02) | 0.01(-0.001, 0.03) |
| **High-fat dairy products** |  |  |  |  |  |
| Demographic, lifestyle, energy^2^ | 0.03(0.004, 0.05) | 0.06(0.03, 0.08) | 0.004(-0.002, 0.01) | 0.04(0.02, 0.06) | 0.01(-0.01, 0.03) |
| + Diet^3^ | 0.02(-0.002, 0.05) | 0.05(0.03, 0.08) | 0.005(-0.002, 0.01) | 0.04(0.02, 0.06) | 0.01(-0.01, 0.03) |
| + BMI^4^ | 0.02(-0.01, 0.04) | 0.05(0.02, 0.07) | 0.01(-0.001, 0.01) | 0.04(0.02, 0.06) | 0.001(-0.02, 0.02) |
| **Low-fat dairy products** |  |  |  |  |  |
| Demographic, lifestyle, energy^2^ | -0.02(-0.05, -0.002) | -0.03(-0.06, -0.01) | -0.002(-0.01, 0.004) | -0.03(-0.05, -0.01) | 0.002(-0.02, 0.02) |
| + Diet^3^ | -0.02(-0.05, 0.001) | -0.03(-0.05, -0.01) | -0.001(-0.01, 0.01) | -0.03(-0.05, -0.01) | 0.004(-0.02, 0.02) |
| + BMI^4^ | -0.02(-0.04, 0.01) | -0.03(-0.05, -0.003) | -0.002(-0.01, 0.01) | -0.03(-0.05, -0.01) | 0.01(-0.01, 0.03) |

^1^Servings as defined by Food Standards Agency 2002: Milk- 1 average glass (200g); Yogurt- 125g carton; Cheese- medium serving (40g); Single cream- 1 tablespoon (15g); Double cream- 1 tablespoon (30g); Butter- 1 teaspoon (10g); Ice-cream- 1 average scoop/tub (60g)

^2^Linear regression model 1: age (years), sex, educational level (low, medium, high), age at completion of full-time education (years), marital status (single, married, widowed or separated), socio-economic status based on occupation (low: technical/semi-routine and routine occupations medium: lower managerial / intermediate occupations; high: professional / higher managerial occupations), individual follow-up time (years), physical activity level (inactive, moderately inactive, moderately active, active), smoking status (never, former and current smoker), lipid-lowering medication (Yes, No), anti-hypertensive medication (Yes, No), hormone-replacement therapy (Yes, No, Men), total energy intake (kcal/day)

^3^Linear regression model 2: Model 1 + intakes (g/d) of fruit, vegetables, potatoes, legumes, nuts, processed cereals, whole-grain cereals, poultry and eggs, red meat, processed meat, fish, sauces, margarine, sweet snacks, sugar-sweetened beverages, artificially sweetened beverages, fruit juice, coffee, tea and alcoholic beverages and dietary supplement use (Yes, No)

^4^Linear regression model 3: Model 2 + BMI (kg/m^2^)

EPIC: European Prospective Investigation into Cancer and Nutrition; UK: United Kingdom

| **Supplementary Table 3** (continued on HbA1c, blood pressure, and metabolic risk z-score) | | | | | |
| --- | --- | --- | --- | --- | --- |
|  | **HbA1c (mmol/mol**) | | **Systolic blood pressure (mmHg)** | **Diastolic blood pressure (mmHg)** | **Metabolic risk z-score** |
| **Mean ± SD of change** | 1.6 ± 6.1 | | 0.5 ± 14.9 | -0.1 ± 10.5 | 0.01 ± 0.34 |
| **Participants (n)** | 6,224 | | 14,210 | 14,231 | 6,033 |
| **Dairy consumption (servings/d)^1^** | b (95% CI) | b (95% CI) | | b (95% CI) | b (95% CI) |
| **Milk** |  |  | |  |  |
| Demographic, lifestyle, energy^2^ | 0.07(-0.18, 0.31) | -0.08(-0.52, 0.36) | | 0.11(-0.21, 0.42) | 0.002(-0.02, 0.02) |
| + Diet^3^ | 0.10(-0.14, 0.35) | -0.10(-0.55, 0.35) | | 0.10(-0.22, 0.43) | 0.003(-0.02, 0.02) |
| + BMI^4^ | 0.10(-0.14, 0.34) | -0.13(-0.56, 0.31) | | 0.09(-0.24, 0.41) |  |
| **Full-fat milk** |  |  | |  |  |
| Demographic, lifestyle, energy^2^ | 0.54(0.08, 1.00) | 0.10(-0.79, 0.99) | | 0.24(-0.40, 0.87) | 0.02(-0.01, 0.05) |
| + Diet^3^ | 0.55(0.09, 1.01) | 0.07(-0.83, 0.96) | | 0.20(-0.43, 0.83) | 0.02(-0.02, 0.05) |
| + BMI^4^ | 0.52(0.06, 0.97) | -0.10(-0.98, 0.78) | | 0.07(-0.54, 0.69) |  |
| **Low-fat milk** |  |  | |  |  |
| Demographic, lifestyle, energy^2^ | 0.00(-0.26, 0.27) | -0.05(-0.62, 0.52) | | 0.12(-0.25, 0.49) | 0.002(-0.02, 0.02) |
| + Diet^3^ | 0.04(-0.23, 0.31) | -0.05(-0.62, 0.51) | | 0.12(-0.25, 0.49) | 0.003(-0.02, 0.02) |
| + BMI^4^ | 0.04(-0.23, 0.31) | -0.09(-0.65, 0.48) | | 0.10(-0.26, 0.47) |  |
| **Yogurt** |  |  | |  |  |
| Demographic, lifestyle, energy^2^ | 0.10(-0.40, 0.61) | -0.16(-1.03, 0.70) | | -0.38(-1.03, 0.27) | -0.01(-0.05, 0.02) |
| + Diet^3^ | 0.16(-0.35, 0.66) | -0.09(-0.98, 0.81) | | -0.29(-0.96, 0.38) | -0.01(-0.04, 0.03) |
| + BMI^4^ | 0.21(-0.29, 0.71) | 0.14(-0.71, 0.98) | | -0.14(-0.78, 0.50) |  |
| **Full-fat yogurt** |  |  | |  |  |
| Demographic, lifestyle, energy^2^ | -0.06(-1.70, 1.59) | 1.33(-1.77, 4.43) | | 0.14(-1.95, 2.23) | 0.02(-0.08, 0.13) |
| + Diet^3^ | -0.02(-1.67, 1.64) | 1.32(-1.80, 4.44) | | 0.08(-2.04, 2.20) | 0.03(-0.07, 0.14) |
| + BMI^4^ | -0.01(-1.68, 1.66) | 1.29(-1.75, 4.32) | | 0.05(-2.02, 2.13) |  |
| **Low-fat yogurt** |  |  | |  |  |
| Demographic, lifestyle, energy^2^ | 0.22(-0.36, 0.80) | -0.20(-1.04, 0.65) | | -0.37(-0.95, 0.22) | -0.02(-0.06, 0.02) |
| + Diet^3^ | 0.26(-0.30, 0.82) | -0.12(-0.97, 0.72) | | -0.27(-0.87, 0.33) | -0.01(-0.05, 0.03) |
| + BMI^4^ | 0.33(-0.23, 0.89) | 0.16(-0.66, 0.99) | | -0.07(-0.66, 0.51) |  |
| **Cheese** |  |  | |  |  |
| Demographic, lifestyle, energy^2^ | -0.11(-0.67, 0.46) | -0.04(-0.94, 0.86) | | -0.15(-0.79, 0.49) | -0.03(-0.06, 0.01) |
| + Diet^3^ | -0.08(-0.64, 0.48) | -0.01(-0.92, 0.89) | | -0.13(-0.78, 0.51) | -0.02(-0.06, 0.01) |
| + BMI^4^ | -0.04(-0.60, 0.52) | 0.08(-0.80, 0.96) | | -0.07(-0.69, 0.56) |  |
| **High-fat cheese** |  |  | |  |  |
| Demographic, lifestyle, energy^2^ | -0.08(-0.81, 0.65) | 1.33(0.00, 2.65) | | 0.75(-0.21, 1.70) | 0.01(-0.05, 0.06) |
| + Diet^3^ | -0.05(-0.76, 0.67) | 1.27(-0.05, 2.60) | | 0.67(-0.28, 1.62) | 0.01(-0.05, 0.06) |
| + BMI^4^ | -0.11(-0.81, 0.60) | 0.83(-0.44, 2.10) | | 0.36(-0.54, 1.26) |  |
| **Low-fat cheese** |  |  | |  |  |
| Demographic, lifestyle, energy^2^ | -0.13(-1.00, 0.73) | -1.26(-2.84, 0.31) | | -1.06(-2.23, 0.10) | -0.04(-0.10, 0.03) |
| + Diet^3^ | -0.09(-0.96, 0.79) | -1.19(-2.78, 0.39) | | -0.98(-2.18, 0.22) | -0.03(-0.10, 0.03) |
| + BMI^4^ | 0.02(-0.86, 0.90) | -0.63(-2.17, 0.90) | | -0.58(-1.77, 0.61) |  |
| **Fermented dairy products** |  |  | |  |  |
| Demographic, lifestyle, energy^2^ | -0.03(-0.38, 0.32) | -0.27(-0.95, 0.41) | | -0.37(-0.83, 0.08) | -0.03(-0.05, -0.004) |
| + Diet^3^ | 0.00(-0.36, 0.35) | -0.24(-0.94, 0.47) | | -0.33(-0.79, 0.14) | -0.02(-0.04, 0.001) |
| + BMI^4^ | 0.04(-0.32, 0.39) | -0.06(-0.74, 0.63) | | -0.20(-0.64, 0.24) |  |
| **Butter** |  |  | |  |  |
| Demographic, lifestyle, energy^2^ | 0.02(-0.22, 0.27) | 0.37(-0.03, 0.76) | | 0.23(-0.08, 0.53) | -0.002(-0.02, 0.01) |
| + Diet^3^ | 0.01(-0.24, 0.25) | 0.35(-0.07, 0.76) | | 0.22(-0.09, 0.53) | -0.001(-0.02, 0.02) |
| + BMI^4^ | 0.00(-0.24, 0.24) | 0.30(-0.12, 0.72) | | 0.19(-0.12, 0.51) |  |
| **Ice-cream** |  |  | |  |  |
| Demographic, lifestyle, energy^2^ | 0.12(-0.58, 0.82) | -0.26(-1.41, 0.89) | | -0.36(-1.21, 0.50) | -0.002(-0.05, 0.05) |
| + Diet^3^ | 0.14(-0.55, 0.84) | -0.35(-1.50, 0.81) | | -0.35(-1.22, 0.52) | -0.003(-0.05, 0.05) |
| + BMI^4^ | 0.14(-0.55, 0.84) | -0.48(-1.62, 0.65) | | -0.43(-1.27, 0.41) |  |
| **Total dairy products** |  |  | |  |  |
| Demographic, lifestyle, energy^2^ | 0.01(-0.14, 0.15) | -0.01(-0.23, 0.22) | | 0.00(-0.16, 0.16) | -0.002(-0.01, 0.01) |
| + Diet^3^ | 0.03(-0.12, 0.17) | -0.01(-0.24, 0.22) | | 0.003(-0.16, 0.17) | 0.00(-0.01, 0.01) |
| + BMI^4^ | 0.03(-0.12, 0.17) | -0.001(-0.23, 0.22) | | 0.01(-0.15, 0.18) |  |
| **High-fat dairy products** |  |  | |  |  |
| Demographic, lifestyle, energy^2^ | 0.06(-0.15, 0.26) | 0.22(-0.11, 0.55) | | 0.18(-0.03, 0.39) | -0.002(-0.01, 0.01) |
| + Diet^3^ | 0.06(-0.15, 0.27) | 0.19(-0.15, 0.54) | | 0.16(-0.06, 0.38) | -0.001(-0.01, 0.01) |
| + BMI^4^ | 0.04(-0.17, 0.25) | 0.11(-0.23, 0.46) | | 0.11(-0.12, 0.34) |  |
| **Low-fat dairy products** |  |  | |  |  |
| Demographic, lifestyle, energy^2^ | 0.01(-0.17, 0.19) | -0.11(-0.43, 0.21) | | -0.04(-0.27, 0.20) | -0.01(-0.02, 0.01) |
| + Diet^3^ | 0.04(-0.14, 0.23) | -0.09(-0.42, 0.24) | | -0.01(-0.24, 0.23) | -0.004(-0.02, 0.01) |
| + BMI^4^ | 0.06(-0.12, 0.25) | -0.01(-0.33, 0.31) | | 0.05(-0.18, 0.28) |  |

^1^Servings as defined by Food Standards Agency 2002: Milk- 1 average glass (200g); Yogurt- 125g carton; Cheese- medium serving (40g); Single cream- 1 tablespoon (15g); Double cream- 1 tablespoon (30g); Butter- 1 teaspoon (10g); Ice-cream- 1 average scoop/tub (60g)

^2^Linear regression model 1: age (years), sex, educational level (low, medium, high), age at completion of full-time education (years), marital status (single, married, widowed or separated), socio-economic status based on occupation (low: technical/semi-routine and routine occupations medium: lower managerial / intermediate occupations; high: professional / higher managerial occupations), individual follow-up time (years), physical activity level (inactive, moderately inactive, moderately active, active), smoking status (never, former and current smoker), lipid-lowering medication (Yes, No), anti-hypertensive medication (Yes, No), hormone-replacement therapy (Yes, No, Men), total energy intake (kcal/day)

^3^Linear regression model 2: Model 1 + intakes (g/d) of fruit, vegetables, potatoes, legumes, nuts, processed cereals, whole-grain cereals, poultry and eggs, red meat, processed meat, fish, sauces, margarine, sweet snacks, sugar-sweetened beverages, artificially sweetened beverages, fruit juice, coffee, tea and alcoholic beverages and dietary supplement use (Yes, No)

^4^Linear regression model 3: Model 2 + BMI (kg/m^2^)

EPIC: European Prospective Investigation into Cancer and Nutrition; HbA1c: Haemoglobin A1c; UK: United Kingdom

| **Supplementary Table 4**. Stratified associations of the change in total and types of dairy products with the change in cardio-metabolic markers m baseline to the first follow-up: analysis stratified by significant effect modifiers in the EPIC-Norfolk study, UK^1^ | | | | | |
| --- | --- | --- | --- | --- | --- |
| **Dairy consumption (servings/d)^2^** | **Cardio-metabolic marker** | **Strata** | **Participants (n)** | **Association**  **b (95% CI)** | ***P***  **interaction** |
| Interaction by age |  |  |  |  |  |
| High-fat cheese | Weight (kg) | < 50 years | 2,795 | 0.47(-0.38, 1.31) |  |
|  |  | 50-60 years | 4,621 | 0.87(0.33, 1.41) |  |
|  |  | ≥60 years | 6,494 | 0.09(-0.39, 0.56) | 0.037 |
| High-fat cheese | BMI (kg/m2) | < 50 years | 2,803 | 0.18(-0.11, 0.47) |  |
|  |  | 50-60 years | 4,620 | 0.28(0.08, 0.48) |  |
|  |  | ≥60 years | 6,506 | 0.04(-0.13, 0.21) | 0.027 |
| Low-fat dairy products | Waist (cm) | < 50 years | 2,830 | -0.16(-0.39, 0.07) |  |
|  |  | 50-60 years | 4,665 | -0.12(-0.29, 0.05) |  |
|  |  | ≥60 years | 6,493 | 0.05(-0.10, 0.21) | 0.019 |
| Interaction by sex |  |  |  |  |  |
| Low-fat milk | Total / HDL cholesterol | Men | 5,404 | -0.06(-0.10, -0.02) |  |
|  |  | Women | 7,263 | 0.02(-0.02, 0.05) | 0.020 |
| Low-fat yogurt | Waist to Hip ratio | Men | 6,087 | -0.003(-0.01, 0.00) |  |
|  |  | Women | 7,852 | 0.001(0.00, 0.00) | 0.027 |
| Total cheese | Waist (cm) | Men | 6,027 | 0.21(-0.32, 0.75) |  |
|  |  | Women | 7,842 | -0.48(-0.88, -0.08) | 0.009 |
| High-fat cheese | Waist (cm) | Men | 6,069 | 0.43(-0.23, 1.08) |  |
|  |  | Women | 7,953 | -0.31(-0.85, 0.22) | 0.021 |
| Interaction by BMI |  |  |  |  |  |
| Yogurt | Total cholesterol (mmol/L) | Normal | 5,579 | -0.07(-0.15, 0.01) |  |
|  |  | Obese/overweight | 7,495 | -0.07(-0.13, 0.003) | 0.014 |
| Yogurt | LDL cholesterol (mmol/L) | Normal | 5,518 | -0.01(-0.09, 0.06) |  |
|  |  | Obese/overweight | 7,179 | -0.01(-0.07, 0.05) | 0.011 |
| Butter | Waist (cm) | Normal | 5,880 | 0.06 (-0.16, 0.28) |  |
|  |  | Obese/overweight | 8,047 | 0.13 (-0.06, 0.31) | 0.003 |
| Butter | Waist / Hip circumference | Normal | 5,859 | 0.0003(-0.002, 0.002) |  |
|  |  | Obese/overweight | 8,055 | 0.001(-0.001, 0.29) | 0.032 |
| Fermented dairy products | Total cholesterol (mmol/L) | Normal | 5,575 | -0.03(-0.09, 0.02) |  |
|  |  | Obese/overweight | 7,474 | 0.003(-0.05, 0.05) | 0.028 |
| Total dairy products | Waist (cm) | Normal | 5,899 | -0.0003(-0.11, 0.11) |  |
|  |  | Obese/overweight | 8,064 | -0.01(-0.12, 0.1) | 0.005 |
| High-fat dairy products | Waist (cm) | Normal | 5,832 | -0.07(-0.21, 0.07) |  |
|  |  | Obese/overweight | 8,002 | 0.08(-0.05, 0.22) | 0.040 |
| ^1^Associations from the maximally adjusted linear regression models are presented, which include: age (years), sex, educational level (low, medium, high), age at completion of full-time education (years), marital status (single, married, widowed or separated), socio-economic status based on occupation (low: technical/semi-routine and routine occupational medium: lower managerial / intermediate occupational; high: professional / higher managerial occupations), individual follow-up time (years), physical activity level (inactive, moderately inactive, moderately active, active), smoking status (never, former and current smoker), lipid-lowering medication (Yes, No), anti-hypertensive medication (Yes, No), hormone-replacement therapy (Yes, No, Men), total energy intake (kcal/day), intakes (g/d) of fruit, vegetables, potatoes, legumes, nuts, processed cereals, whole-grain cereals, poultry and eggs, red meat, processed meat, fish, sauces, margarine, sweet snacks, sugar-sweetened beverages, artificially sweetened beverages, fruit juice, coffee, tea and alcoholic beverages, dietary supplement use (Yes, No) and BMI (kg/m2; in associations other than those for metabolic risk z-score). When repeated measures of the covariates were available at baseline and first follow-up, their change was also included. | | | | | |
| ^2^Servings as defined by Food Standards Agency 2002: Milk- 1 average glass (200g); Yogurt- 125g carton; Cheese- medium serving (40g); Single cream- 1 tablespoon (15g); Double cream- 1 tablespoon (30g); Butter- 1 teaspoon (10g); Ice-cream- 1 average scoop/tub (60g) | | | | | |
| EPIC: European Prospective Investigation into Cancer and Nutrition ;UK: United Kingdom | | | | | |

| **Supplementary Table 5**. Associations of the repeated measures of total and types of dairy products at baseline (1993-1997) and first follow-up (1998-2000) with the repeated measures of lipid markers at the first and the second (2004-2011) follow-up in the EPIC-Norfolk study, UK^1^ | | | | | | | | | |
| --- | --- | --- | --- | --- | --- | --- | --- | --- | --- |
|  | **Total / HDL-C** | **Total cholesterol (mmol/l)** | | **HDL-C (mmol/l)** | | **LDL-C (mmol/l)** | | **Triglycerides (mmol/l)** | |
| **Mean ± SD at first follow-up** | 4.4 **±** 1.6 | 6.1 **±** 1.2 | | 1.5 **±** 0.5 | | 3.8 **±** 1.0 | | 1.9 **±** 1.1 | |
| **Mean ± SD at second follow-up** | 3.7 **±** 1.1 | 5.4 **±** 1.1 | | 1.5 **±** 0.4 | | 3.2 **±** 1.0 | | 1.7 **±** 0.9 | |
| **Participants (n)** | 13 307 | 13 557 | | 13 260 | | 13 283 | | 13 530 | |
| **Dairy consumption (servings/d)** | b (95% CI) | b | 95% CI | b | 95% CI | b | 95% CI | b | 95% CI |
| Milk | 1.18* (0.64, 1.72) | -0.01 (-0.03, 0.02) | | -0.02* (-0.03, -0.01) | | 0.00 (-0.02, 0.02) | | 1.34* (0.71, 1.99) | |
| Full-fat milk | 1.59* (0.84, 2.34) | 0.04 (0.01, 0.08) | | -0.01 (-0.03, 0.00) | | 0.03 (0.00, 0.06) | | 1.92* (1.02, 2.83) | |
| Low-fat milk | 1.14* (0.61, 1.67) | -0.01 (-0.03, 0.02) | | -0.02* (-0.03, -0.01) | | 0.00 (-0.02, 0.02) | | 1.28* (0.63, 1.93) | |
| Yoghurt | -0.39 (-1.35, 0.58) | 0.00 (-0.04, 0.04) | | 0.01 (-0.01, 0.02) | | 0.00 (-0.04, 0.04) | | -0.26 (-1.38, 0.86) | |
| Full-fat yoghurt | -1.63 (-4.43, 1.24) | 0.12 (-0.01, 0.26) | | 0.06 (0.02, 0.10) | | 0.07 (-0.06, 0.20) | | -0.53 (-3.77, 2.82) | |
| Low-fat yoghurt | -0.23 (-1.22, 0.77) | -0.01 (-0.06, 0.04) | | 0.00 (-0.01, 0.02) | | 0.00 (-0.04, 0.04) | | -0.24 (-1.39, 0.92) | |
| Cheese | -0.30 (-1.33, 0.74) | 0.00 (-0.05, 0.05) | | 0.01 (0.00, 0.03) | | -0.01 (-0.05, 0.04) | | -1.35 (-2.55, -0.13) | |
| High-fat cheese | -1.86 (-3.23, -0.47) | -0.03 (-0.09, 0.04) | | 0.03 (0.01, 0.05) | | -0.05 (-0.11, 0.01) | | -2.15 (-3.73, -0.53) | |
| Low-fat cheese | 1.33 (-0.11, 2.78) | 0.03 (-0.04, 0.09) | | 0.00 (-0.03, 0.02) | | 0.03 (-0.02, 0.09) | | -0.48 (-2.15, 1.23) | |
| Fermented dairy products | -0.34 (-1.03, 0.34) | 0.00 (-0.03, 0.03) | | 0.01 (0.00, 0.02) | | 0.00 (-0.03, 0.03) | | -0.78 (-1.59, 0.03) | |
| Butter | 0.22 (-0.28, 0.72) | 0.04* (0.02, 0.07) | | 0.01 (0.00, 0.01) | | 0.04* (0.01, 0.06) | | -0.06 (-0.65, 0.54) | |
| Ice-cream | 2.92* (1.52, 4.34) | 0.06 (0.00, 0.13) | | -0.02 (-0.04, 0.00) | | 0.08 (0.02, 0.14) | | 1.21 (-0.38, 2.83) | |
| Total dairy products | 0.56 (0.16, 0.96) | 0.02 (0.00, 0.04) | | 0.00 (-0.01, 0.00) | | 0.02 (0.00, 0.03) | | 0.34 (-0.12, 0.81) | |
| High-fat dairy products | 0.31 (-0.11, 0.73) | 0.04 (0.01, 0.06) | | 0.00 (0.00, 0.01) | | 0.03 (0.01, 0.04) | | 0.19 (-0.30, 0.69) | |
| Low-fat dairy products | 0.79 (0.32, 1.27) | 0.00 (-0.02, 0.02) | | -0.01* (-0.02, -0.01) | | 0.00 (-0.02, 0.02) | | 0.76 (0.20, 1.32) | |
| ^1^ Associations from the maximally adjusted linear mixed models are presented, which include: age (years), sex, educational level (low, medium, high), age at completion of full-time education (years), marital status (single, married, widowed or separated), socio-economic status based on occupation (low: technical/semi-routine and routine occupations; medium: lower managerial / intermediate occupations; high: professional / higher managerial occupations), individual follow-up time (years), physical activity level (inactive, moderately inactive, moderately active, active), smoking status (never, former and current smoker), lipid-lowering medication (Yes, No), anti-hypertensive medication (Yes, No), hormone-replacement therapy (Yes, No, Men), total energy intake (kcal/day), intakes (g/d) of fruit, vegetables, potatoes, legumes, nuts, processed cereals, whole-grain cereals, poultry and eggs, red meat, processed meat, fish, sauces, margarine, sweet snacks, sugar-sweetened beverages, artificially sweetened beverages, fruit juice, coffee, tea and alcoholic beverages, dietary supplement use (Yes, No) and BMI (kg/m2). When repeated measures of the covariates were available at baseline and first follow-up, they were also used. | | | | | | | | | |
| ^2^Servings as defined by Food Standards Agency 2002: Milk- 1 average glass (200g); Yoghurt- 125g carton; Cheese- medium serving (40g); Single cream- 1 tablespoon (15g); Double cream- 1 tablespoon (30g); Butter- 1 teaspoon (10g); Ice-cream- 1 average scoop/tub (60g) | | | | | | | | | |
| *P< 9x10^-4^, which is the cut-off point as derived after False discovery rate correction | | | | | | | | | |
| EPIC: European Prospective Investigation into Cancer and Nutrition ;UK: United Kingdom | | | | | | | | | |

| **Supplementary Table 6**. Longitudinal associations of the repeated measures of total and types of dairy products at baseline (1993-1997) and first follow-up (1998-2000) with the repeated measures of the markers of body weight and composition at the first and the second (2004-2011) follow-up in the EPIC-Norfolk study, UK^1^ | | | | | |
| --- | --- | --- | --- | --- | --- |
|  | **Weight (kg)** | **BMI (kg/m**^2^) ^2^ | **Waist (cm)** | **Waist / Hip circumference** | **Body fat (%)** |
| **Mean ± SD at first follow-up** | 73 **±** 12.8 | 26.7 **±** 4.0 | 88 **±** 12.5 | 0.8 **±** 0.1 | 32.7 **±** 11.3 |
| **Mean ± SD at second follow-up** | 74 **±** 13.2 | 26.8 **±** 4.3 | 94.2 **±** 12.2 | 0.9 **±** 0.1 | 31.6 **±** 8.1 |
| **Participants (n)** | 14,145 | 14,107 | 14,199 | 14,218 | 14,019 |
| **Dairy consumption (servings/d)^3^** | b (95% CI) | b (95% CI) | b (95% CI) | b (95% CI) | b (95% CI) |
| Milk | 0.16* (0.07, 0.26) | 0.12* (0.06, 0.19) | 0.01 (-0.13, 0.15) | -0.001 (-0.002, 0.00) | 0.10 (-0.02, 0.21) |
| Full-fat milk | 0.09 (-0.04, 0.22) | -0.01 (-0.09, 0.07) | -0.28 (-0.48, -0.08) | -0.004* (-0.006, -0.002) | -0.04 (-0.18, 0.11) |
| Low-fat milk | 0.16 (0.07, 0.25) | 0.13* (0.08, 0.19) | 0.01 (-0.13, 0.15) | -0.001 (-0.002, 0.00) | 0.12 (0.02, 0.23) |
| Yoghurt | 0.32* (0.15, 0.49) | 0.27* (0.17, 0.37) | 0.54* (0.28, 0.79) | 0.002 (0.00, 0.004) | 0.22 (0.03, 0.41) |
| Full-fat yoghurt | -0.01 (-0.51, 0.50) | -0.17 (-0.46, 0.11) | -0.06 (-0.82, 0.70) | -0.003 (-0.009, 0.004) | -0.19 (-0.77, 0.38) |
| Low-fat yoghurt | 0.35* (0.18, 0.53) | 0.31* (0.21, 0.41) | 0.59* (0.33, 0.85) | 0.002 (0.00, 0.004) | 0.25 (0.06, 0.45) |
| Cheese | 0.05 (-0.12, 0.23) | 0.00 (-0.10, 0.11) | -0.16 (-0.43, 0.10) | -0.002 (-0.004, 0.00) | -0.22 (-0.43, -0.02) |
| High-fat cheese | -0.22 (-0.47, 0.03) | -0.18 (-0.33, -0.03) | -0.42 (-0.79, -0.05) | -0.005* (-0.008, -0.002) | -0.77 (-1.06, -0.48) |
| Low-fat cheese | 0.33 (0.09, 0.58) | 0.19 (0.04, 0.34) | 0.11 (-0.26, 0.48) | 0.001 (-0.002, 0.004) | 0.36 (0.08, 0.64) |
| Fermented dairy products | 0.20 (0.07, 0.32) | 0.15* (0.08, 0.22) | 0.21 (0.02, 0.39) | 0.00 (-0.002, 0.002) | 0.01 (-0.13, 0.15) |
| Butter | -0.01 (-0.10, 0.08) | 0.00 (-0.05, 0.05) | -0.12 (-0.25, 0.02) | -0.001 (-0.002, 0.00) | 0.03 (-0.07, 0.13) |
| Ice-cream | 0.11 (-0.13, 0.35) | 0.24 (0.09, 0.39) | -0.22 (-0.59, 0.14 | -0.002 (-0.005, 0.001) | 0.39 (0.12, 0.66) |
| Total dairy products | 0.10 (0.03, 0.17) | 0.08 (0.03, 0.12) | -0.01 (-0.11, 0.09) | -0.001 (-0.002, 0.00) | 0.07 (-0.02, 0.15) |
| High-fat dairy products | 0.01 (-0.07, 0.09) | -0.03 (-0.07, 0.01) | -0.17 (-0.28, -0.05) | -0.002* (-0.003, -0.001) | -0.05 (-0.13, 0.04) |
| Low-fat dairy products | 0.19* (0.11, 0.28) | 0.16* (0.10, 0.21) | 0.09 (-0.03, 0.21) | -0.001 (-0.002, 0.00) | 0.12 (0.02, 0.22) |
| ^1^ Associations from the maximally adjusted linear mixed models are presented, which include: age (years), sex, educational level (low, medium, high), age at completion of full-time education (years), marital status (single, married, widowed or separated), socio-economic status based on occupation (low: technical/semi-routine and routine occupations medium: lower managerial / intermediate occupations; high: professional / higher managerial occupations), individual follow-up time (years), physical activity level (inactive, moderately inactive, moderately active, active), smoking status (never, former and current smoker), lipid-lowering medication (Yes, No), anti-hypertensive medication (Yes, No), hormone-replacement therapy (Yes, No, Men), total energy intake (kcal/day), intakes (g/d) of fruit, vegetables, potatoes, legumes, nuts, processed cereals, whole-grain cereals, poultry and eggs, red meat, processed meat, fish, sauces, margarine, sweet snacks, sugar-sweetened beverages, artificially sweetened beverages, fruit juice, coffee, tea and alcoholic beverages, dietary supplement use (Yes, No) and BMI (kg/m2; in associations other than those for weight and BMI). When repeated measures of the covariates were available at baseline and first follow-up, they were also used. | | | | | |
| ^2^ BMI: Body mass index | | | | | |
| ^3^ Servings as defined by Food Standards Agency 2002: Milk- 1 average glass (200g); Yoghurt- 125g carton; Cheese- medium serving (40g); Single cream- 1 tablespoon (15g); Double cream- 1 tablespoon (30g); Butter- 1 teaspoon (10g); Ice-cream- 1 average scoop/tub (60g) | | | | | |
| *P< 9x10^-4^, which is the cut-off point as derived after False discovery rate correction | | | | | |
| EPIC: European Prospective Investigation into Cancer and Nutrition ;UK: United Kingdom | | | | | |
